# USP24 promotes drug resistance during cancer therapy

**DOI:** 10.1038/s41418-021-00778-z

**Published:** 2021-04-12

**Authors:** Shao-An Wang, Ming-Jer Young, Yi-Chang Wang, Shu-Hui Chen, Chia-Yu Liu, Yao-An Lo, Hung-Hsiang Jen, Kai-Cheng Hsu, Jan-Jong Hung

**Affiliations:** 1 Department of Biotechnology and Bioindustry Sciences, National Cheng Kung University, Tainan, Taiwan; 2 School of Respiratory Therapy, College of Medicine, Taipei Medical University, Taipei, Taiwan; 3 Department of Chemistry, College of Science, National Cheng Kung University, Tainan, Taiwan; 4 Graduate Institute of Cancer Biology and Drug Discovery, College of Medical Science and Technology, Taipei Medical University, Taipei, Taiwan; 5 Graduate Institute of Medical Sciences, College of Medicine, Taipei Medical University, Taipei, Taiwan

**Keywords:** Translational research, Deubiquitylating enzymes, Drug development

## Abstract

Drug resistance has remained an important issue in the treatment and prevention of various diseases, including cancer. Herein, we found that USP24 not only repressed DNA-damage repair (DDR) activity by decreasing Rad51 expression to cause the tumor genomic instability and cancer stemness, but also increased the levels of the ATP-binding cassette (ABC) transporters P-gp, ABCG2, and ezrin to enhance the pumping out of Taxol from cancer cells, thus resulted in drug resistance during cancer therapy. A novel USP24 inhibitor, NCI677397, was screened for specific inhibiting the catalytic activity of USP24. This inhibitor was identified to suppress drug resistance via decreasing genomic instability, cancer stemness, and the pumping out of drugs from cancer cells. Understanding the role and molecular mechanisms of USP24 in drug resistance will be beneficial for the future development of a novel USP24 inhibitor. Our studies provide a new insight of USP24 inhibitor for clinically implication of blocking drug resistance during chemotherapy.

## Introduction

Deubiquitinases (DUBs) are specific enzymes that regulate multiple cellular functions by modulating ubiquitin molecules. Ubiquitin-specific peptidases belong to the super family of DUB superfamily, which has been correlated with many human disease processes, including cancer progression [1–3]. More than 50 ubiquitin-specific peptidases have been identified, and most of these enzymes exert their functions by reversing the polyubiquitination or monoubiquitination of target proteins [4–6]. Malfunction of the ubiquitin system, which has been implicated in the tumorigenesis of various cancers, can either enhance the effect of oncogenes or reduce the activity of tumor-suppressor genes [7, 8]. USP24 is a 2620-amino acid protein that contains one ubiquitin-associated domain (UBA), which binds to the ubiquitin molecule on substrate proteins, and one ubiquitin C-terminal hydrolase domain, which is the catalytic domain. Previous studies indicate that two single nucleotide polymorphisms (SNPs) are found in USP24, which have been implicated in Parkinson’s disease [7, 9–11]. In our previous study, we demonstrated that USP24 expression was upregulated in most late-stage lung cancer patients due to increasing mRNA stability caused by SNPs or RNA editing [12]. Recently, we also found that USP24 is upregulated in tumor-associated macrophages to promote cancer malignancy [13].

DNA damage is triggered under various conditions and can be divided into double-stranded DNA damage and single-stranded DNA damage [14]. Various DNA-damage repair (DDR) pathways, including nonhomologous end joining, homologous recombination (HR), and DNA mismatch repair [15], are triggered by different conditions. Loss of the ability to repair DNA damage will cause mutations, which results in the induction of drug resistance in cancer cell lines under chemotherapy or targeted therapy. Lung cancers, including small-cell lung cancer (SCLC) and non-small-cell lung cancer, are characterized by a high mutational burden in genes encoding proteins such as EGFR, Kras, TP53, ALK, and MYC, which promotes genomic instability [16]. In addition, cancer cell metabolism is involved in DNA damage and repair mechanisms through the regulation of methyl- and acetyl-group donors, de novo nucleotide synthesis and reactive oxygen species, which can increase oxidative DNA damage and the load on the DNA repair machinery [17].

Drug resistance is induced by drug treatment for various diseases, such as bacterial infection and cancer, which decreases the effectiveness of the drug. One major challenge is the multifactorial nature of resistance. Many factors, including increased drug efflux, decreased drug uptake, altered cell cycle checkpoints, the induction of emergency response genes, apoptosis inhibition, drug compartmentalization and altered drug targets, are involved in drug resistance [18]. Three broad groups of ABC transporters have been implicated in multiple drug resistance (MDR): P-glycoprotein, ABCG2, and MDR-associated proteins (MRPs) [19, 20]. In addition, several related proteins, such as the ezrin, radixin, and moesin (ERM) proteins, can interact with actin to facilitate the membrane localization of P-gp [21]. The other challenge is heterogeneity of tumor cells [22]. Genome stabilization can decrease the rate of mutation, prevent the dysregulation of genes, and can thus potentially inhibit drug resistance during cancer therapy [23]. Various treated strategies induce genomic instability, which might be the major reason for drug resistance [24, 25]. Our results in this study indicated that USP24 can promote drug resistance during cancer therapy by stabilizing several ABC transporters and inducing genomic instability.

## Material and methods

### Cell culture and transfection

Human lung adenocarcinoma epithelial cell line A549 and human nasopharyngeal carcinoma (NPC) cell line HONE-1 were obtained from the American Type Culture Collection (ATCC) are cultured with RPMI 1640 medium (Life technologies, NY, USA). Human primary glioblastoma (GBM) cell line Pt’3 was obtained according to a protocol approved by the Taipei Medical University Internal Review Board (approval no. 201006011 and 201402018) and human colorectal carcinoma cell line HCT116 was obtained from ATCC cultured in Dulbecco’s modified Eagle’s medium (Thermo Fisher Scientific, Waltham, MA, USA). DMEM and RPMI medium contain 10% fetal bovine serum, 100 μg/ml streptomycin sulfate, and 100 U/ml penicillin G sodium. All cell lines were maintained at 37 °C and 5% CO_2_. Taxol-resistant A549, CDDP-resistant A549, CPT-resistant A549, CPT-resistant HONE-1, OX-resistant HCT116, and temozolomide (TMZ)-resistant GBM cells were maintained in the same culture medium containing Taxol, CDDP, CPT, OX, and TMZ (Sigma-Aldrich, St. Louis, MO, USA), respectively. In order to generate M2 macrophage, THP-1 cells were cultured with RPMI medium containing 50 nM PMA (Merck Millipore, Billerica, MA, USA) for 24 h, and adding 20 ng/ml IL-4 (Peprotech, Rocky Hill, NJ, USA) and 20 ng/m IL-13 (Peprotech, Rocky Hill, NJ, USA) for another 24 h. PolyJet (SignaGen Laboratories, Rockville, MD, USA) was used to transfect expression vectors into cancer cell lines according to manufacturer’s protocol. For spheroid cell culture, cells were cultured in serum-free DMEM/F12 (Thermo Fisher Scientific) supplemented with 10 ng/ml epidermal growth factor and 10 ng/ml fibroblast growth factor (Thermo Fisher Scientific) in ultra-low attachment 6 cm dish or 96-well plate.

### Immunohistochemistry

All human study has been conducted in accordance with the guidelines and regulations. The study using human specimens was approved by the Clinical Research Ethics Committee at National Cheng Kung University Medical Center (Tainan, Taiwan), IRB No.: A-ER-107-367. Human specimens were incubated in 10% formaldehyde for 24 h for fixation, dehydration, and embedded in paraffin. Hematoxylin and eosin were used for staining sections (5 mm). For immunohistochemistry, paraffin-embedded sections were incubated in xylene for dewaxing and a graded series of ethanol for dehydration. Sections were incubated in PBS with 0.3% hydrogen peroxide for 30 min to block endogenous peroxidase, and then incubated in PBS with 1% bovine serum albumin (BSA) for blocking. The anti-USP24 (1:200), anti-CD44 (1:100), and anti-CD133 (1:200) antibodies were used to cover sections for 1 h in room temperature, and Vectastain ABC kit (Vector Laboratories, Burlingame, CA, USA) was used for visualizing the immunoreactivity. Sections were photographed by Olympus BX-51 microscope (Olympus, Melville, NY, USA).

### Immunofluorescent analysis

Scramble and USP24-knockdown lentivirus infected cells were seeded in 6-well plates with cover slips inside for 48 h. Cover slips were removed and cells were fixed with 4% paraformaldehyde in 4 °C for 15 min. After fixation, cover slips were washed with PBS, and incubated with 0.2% Triton X-100 in PBS for 5 min at room temperature. Cover slips were then blocked with 1% BSA for 1 h, and stained with anti-Rad51 antibody (1:200, Genetex), anti-GFP (santa cruz), anti-CENPA (abcam), or anti-γ-H2Ax (abcam) for 16 h at 4 °C. After washing with PBS, cells were stained with Alexa Fluor^®^ 488 or 568 (Invitrogen) for 1 h at room temperature, and mounted with 90% glycerol containing DAPI (Invitrogen). Number of stained cells, Rad51, and γ-H2Ax foci were examined by fluorescence microscopy (Olympus, Tokyo, Japan). ImageJ (Bethesda, Maryland, USA.) were used to perform the statistical analysis of Rad51 and γ-H2Ax foci.

### Transwell migration assay

A549 cells infected with lentivirus or treated with conditioned medium from M2 macrophage for 24 h were suspended in serum-free DMEM or RPMI and seeded into the inserts of transwell chambers at the density of 2 × 10^4^ for 6 h. For transwell migration assay, transwell chamber contained DMEM or RPMI medium with 10% FBS. Invaded cells were stained with DAPI (Invitrogen, Carlsbad, CA, USA) and examined by fluorescence microscopy (Olympus, Tokyo, Japan). ImageJ (Bethesda, Maryland, USA) was used to perform the statistical analysis of invaded cells.

### Homology modeling for USP24 structure

A homology modeling analysis was performed because the USP24 structure was unavailable. First, we identified a template, USP7 (PDB ID: 5N9R), which is a homology protein of USP24 and available on the Protein Data Bank [26]. Next, the USP24 protein sequence was aligned to the template. Finally, a homology model was created using the MODELLER [27] component within the software Chimera [28].

### Xenograft model

All experiments and animal care were conducted in accordance with the guidelines and regulations and the experiments related with animals were approved by the Institutional Animal Care and Use Committee (IACUC) at National Cheng Kung University (NCKU), Taiwan (IACUC approval No. 106223). T24 cells (1 × 10^7^) were infected with scramble and shUSP24 lentivirus in a dose of 5 moi for 24 h, and implanted into the back of severe combined immune-deficient (SCID) mice. After 1 week of implantation, Taxol or NCI677397 treatment was twice every week and tumor volume was detected. Tumors were resected 4 weeks after implantation and weighted.

### LC/MS/MS analysis

In total, 1 μl of the extracted sample was injected into an HPLC column (5 cm × 2.1 mm, 2 μm C18, 50811-U, SUPELCO Corporation, Bellefonte, PA, USA), coupled online to a QE-Orbitrap instrument (Thermo). The sample was eluted at 40 °C using mobile phase A (0.1% formic acid) and mobile phase B (100% methanol) for a 3.25-min isocratic elution under 90% phase B at a flow rate of 0.3 ml/min. The precursor ion of Taxol was set at *m*/*z* = 854.34 and three product ions (*m*/*z* = 286.11, 509.22, and 569.24) were targeted. The MS selection window for the retention time was 1 min and *m*/*z* ≤ 4 for the precursor ion using quadrupole (Q1). The precursor ion was fragmented in the collision cell (Q2) under a collision energy of 20, and all product ions were detected by Orbitrap at a resolution (*R* = 17,500). A linear regression line was constructed based on the ion intensity measured from the eight spiked standards indicated above with *R*^2^ > 0.96. The concentration of the drug in the treated cells was then determined based on the regression equation.

### In vitro deubiquitination assay

A549 cells were treated with MG132 (Sigma-Aldrich) for 12 h. P-gp protein in A549 cell lysates were immunoprecipitated by incubating with anti-P-gp antibody for 4 h, and then incubated with protein A agarose (Millipore) for 1 h. After washing, substrate was mixed with human recombinant USP24 protein (50 μg per ml) (OriGene) in deubiquitination buffer (50 mM Tris PH 8.0, 10 mM DTT and 5 μM MG132) for 2 h in 37 °C. Reaction was stopped by adding sample buffer and signal of ubiquitinated proteins were analyzed by western blotting.

### Structure-based virtual screening for potential USP24 inhibitors

The modeled USP24 structure was prepared using the molecular docking platform LeadIT [29]. The USP24 homology model was aligned to the template structure. The co-crystal ligand of the template structure (USP7) was used to determine the binding site. The binding site was defined as residues within a radius of 10 Å of the co-crystal ligand. Water molecules were removed. The NCI compounds (roughly 250,250 compounds) were docked into the binding site using LeadIT. The docking procedure used a hybrid enthalpy and entropy approach. A maximum number of solution per iteration and fragmentation was set to 200, respectively. In this study, 1,500,000 compounds were used this same docking approach with the ChemDiv compound database.

### Western blotting analysis

Cells were collected by sample buffer and analyzed by electrophoresis. Proteins were transferred to polyvinylidene difluoride (PVDF, Millipore) membrane and TBST buffer (10 mM Tris-HCl, pH 8.0, 150 mM NaCl and 0.05% Tween 20) containing 5% nonfat milk was used for blocking. Anti-USP24 (Proteintech), anti-p300 (BD), anti-actin (Sigma-Aldrich), anti-ubiquitin (Santa Cruz), anti-P-gp (Genetex), anti-ABCG2 (Genetex), anti-MRP1 (Genetex), anti-MRP3 (Genetex), anti-Ezrin (Genetex), lamin A/C (Santa Cruz), anti-γ-H2Ax (abcam), anti-biotin (Genetex), anti-Flag (Genetex), anti-Rad51 (abcam), and anti-BRD7 (Sigma-Aldrich) were used for probing interested proteins. After incubated with primary antibodies, PVDF membranes were then incubated with secondary immunoglobulin antibodies linked with horse radish peroxidase (Millipore, 1:10,000). For detecting immunoprecipitated samples, light chain-specific secondary antibodies were used (Jackson ImmunoResearch, 1:10,000). ECL Western blotting detection system (Millipore) and ChemiDoc-it imager (UVP) were used for detecting signals.

### Lentivirus knockdown system

Scramble knockdown and USP24 knockdown lentivirus were generated from RNAi core facility of Academia Sinica (Taipei, Taiwan). Cells were seeded in 6-well plates and incubated for 16 h, and then treated with 1 ml RPMI medium containing 10 μg Polybrene (Millipore) and lentivirus with 5 moi. After 24 h of infection, medium containing lentivirus was replaced with fresh medium and maintained for another 72 h.

### Genomic DNA extraction

Genomic DNA of human lung specimens were extracted by using QIAamp DNA Mini kit (QIAGEN, Germantown, MD, USA) according to manufacturer’s protocol.

### Immunoprecipitation

Cells were harvested in protein lysis buffer containing protease inhibitor cocktail (Merck Millipore, Bedford, MA, USA). Five hundred microgram of protein was incubated with the anti-USP24 (Proteintech) antibody followed by the precipitation using protein A/G agarose (Merck Millipore). After washing four times by lysis buffer, the complex was mixed with 2X SDS sample buffer containing β-mercaptoethanol (Millipore Sigma Corporate), and subjected to western blotting.

### Cell viability assay

Cells treated with NCI677397, CPT or Oxaliplatin and seeded in 96-well plates (1 × 10^4^ cells/well) for 24 h. Cell viability was examined by using PrestoBlue cell viability kit according to the manufacturer’s instruction (Thermo Fisher Scientific).

### Cytoplasmic, nuclear, and chromatin fractionation

After indicated treatments, 3 × 10^6^ cells were harvested and wash with cold PBS. Cells were then suspended with 400 μl of buffer A (10mMHEPES (pH 7.9), 10 mM KCl, 1.5 mM MgCl2, 0.34 M sucrose, 10% glycerol, 1 mM dithiothreitol, 1 mM PMSF, 10 μM leupeptin, and 0.3 μM aprotinin (Sigma-Aldrich, St. Louis, Missouri, USA)). Cells were incubated on ice for 5 min after adding Triton X-100 (Sigma-Aldrich, St. Louis, Missouri, USA) to a final concentration of 0.1%. Part of the cell suspension were collected as total cell lysates, and cytoplasmic proteins were separated from nuclei by low speed centrifuge (1300 × *g*) for 4 min at 4 °C. Nuclear part were washed with 1 ml buffer A and one part served as total nucleus lysates while the rest were resuspended by 150 μl of buffer B (3 mM EDTA, 0.2 mM EGTA, 1 mM dithiothreitol, 1 mM PMSF, 10 μM leupeptin, and 0.3 μM aprotinin). After incubated on ice for 30 min, insoluble chromatin was separated from soluble nuclear proteins by centrifuge (1700 × *g*) for 4 min at 4 °C.

### Reporter assay

A549 cells were infected with scramble or USP24 shRNA expressing lentivirus for 3 days. Cells were then transfected with different truncated Rad51 reporter plasmid for 24 h in 6-well plate, and luciferase assay was performed by using dual-luciferase reporter assay kit (Promega, Madison, WI, USA) according to manufacturer’s protocol. The reporter activity was normalized by protein concentration eliminate the effect of proliferation.

### cDNA microarray

A549 cells were infected with scramble or shUSP24 expressing lentivirus for 4 days, and total RNA of were extracted by using TRIsure (Bioline, Taunton, MA, USA). Extract RNA were analyzed by using microarray analysis. (Phalanx Biotech, Hsinchu, Taiwan).

### Wound healing assay

In total, 1.4 × 10^4^ A549 cells were cultured in the space of culture inserts of wound healing assay cell culture dish (Ibidi, Martinsried, Germany) for 24 h. Insert was removed and the width between separated cells was examined by microscopy (Olympus, Tokyo, Japan) in indicated time points.

### Protein stability assay

Cells were infected with scramble or USP24 shRNA expressing lentivirus for 4 days, and treated with 100 μg/ml cycloheximide (Sigma-Aldrich, St. Louis, Missouri, USA) to inhibit protein translation. Cells were resolved in sample buffer at indicated time, and protein stability was analyzed by western blot. Protein level was quantified by using Multi Gauge 3.0 software (Fujifilm, Japan).

### Rad51 reporter plasmid construction

Different region of Rad51 promoter sequences (transcription start site as +1; −700 to +300; −366 to +300; −133 to +300; −73 to +300; −13 to +300; +1 to +300; +43 to +300) was cloned into pGL2-basic luciferase reporter vector (Promega, Madison, WI, USA).

### Site-directed mutation

To introduce site-directed mutation into the E2F-binding site of Rad51 promoter sequence and different USP24 mutants, QuikChange Site-Directed Mutagenesis Kit (Agilent Technologies, Santa Clara, CA, USA) was used according to manufacturer’s protocol. Mutagenic PCR primers for site-directed mutagenesis were listed in table.

### RT-PCR

RNA from indicated cells was extracted by using TRIsure RNA extraction kit (Bioline, London, UK), and 3 μg of purified RNA was converted into cDNA by reverse transcription with SuperScript III reverse transcriptase (Invitrogene). PCR was performed by using SuperTherm Taq DNA polymerase (GeneCraft, Köln, Germany) according to manufacturer’s instructions.

### Colony-formation assay

A549 cells were infected with lentivirus expressing scramble and USP24 shRNA for 3 days and seeded in 6-well plates at a density of 5 × 10^3^ per well. After 2 weeks of culture, cells were washed with PBS and colonies were visualized by incubating with 2% Methylene blue (Sigma-Aldrich, St. Louis Missouri, USA) for 30 min. After 30 min of incubation, methylene blue was removed and colonies were washed with distilled water for three times. ImageJ (Bethesda, Maryland, USA) were used to perform the statistical analysis of colony numbers.

### Homologous recombination assay

Stable expressing integrated HR reporter DR-GFP U2OS cells (gift from Dr. Hungjiun Liaw at the Department of Life Sciences, NCKU, Taiwan) were infected with scramble or USP24 shRNA expressing lentivirus for 3 days, and then co-transfected with plasmid expressing I-SecI restriction enzyme and DsRed monomer for 2 days. DsRed monomer plasmid was added in 0.5:3 ratio to mark the I-SceI-positive cells. Cells were harvested and analyzed by flow cytometry, and only successfully transfected mRFP-positive cells were analyzed for HR efficiency. Data were analyzed with CellQuest software to reveal the percentage of GFP-positive cells relative to the mRFP-positive cells. The data were set to 1% of the background level of GFP-positive cells in every internal control (transfected with vector only).

### Chromatin immunoprecipitation assay

A549 cells were infected with scramble or USP24 shRNA expressing lentivirus, and added 37% of formaldehyde (Merck Millipore, Billerica, MA, USA) into medium to a final concentration of 1% at room temperature for 10 min. Cells were washed with PBS and resuspended with lysis buffer (25 nM, pH 7.5 Tris-HCl, 150 mM NaCl, 5 mM EDTA, 1% Triton X-100, 1% SDS). Samples were sonicated (output level of 4, 15 s on and 15 s off, total 3 min) on ice to shear chromatin to an average length between 300 and 500 bps and collect the supernatant by centrifugation with 8000 rpm 2 min at 4 °C. Diluted 50 μl supernatant with 450 μl dilution buffer (50 mM, pH 8.0 Tris-HCl, 0.5% NP-40, 0.2 M NaCl, 0.5 mM EDTA) and anti-E2F4 antibody (1:250, abcam) or Normal IgG (1:250, Santa Cruz) was added. Samples were incubated at 4 °C for 16 h on a rotating device, then added with 20 μl salmon sperm DNA/protein G agarose (Stratagene, LA, CA, USA.) and incubated at 4 °C for another 1 h. Agarose beads were collected by centrifugation at 4000 rpm at 4 °C for 1 min and washed with high salt buffer (20 mM pH 8.0 Tris-HCl, 0.5 % NP-40, 0.5 M NaCl, 2 mM EDTA) for three times and low salt buffer (10 mM pH 8.0 Tris-HCl, 0.5% NP-40, 0.1 M NaCl, 1 mM EDTA, 0.01% SDS) for one time. Beads were resuspended with 1 ml TE buffer (10 mM pH 8.5 Tris-HCl, 0.1 mM EDTA) with 1% SDS, and boiled at 65 °C for 2 h on a heat block. Supernatant was removed boiled at 65 °C for another 16 h. DNA was precipitated by using phenol/chloroform and washed with 70% alcohol. Rad51 promoter was analyzed by PCR.

### Fluorescence-activated cell sorting (FACS)

For cell cycle analysis, A549 cells were infected with scramble or USP24 shRNA expressing lentivirus. On day 4, cells were washed with PBS and fixed with 70% alcohol in 4 °C for 16 h. Cells were then incubated in cold PBS with 0.1% Triton X-100 for 10 min for permeabilization. Permeabilized cells were treated with 10 μg/ml RNase A (Qiagen, Germantown, MD, USA), and 50 μg/ml of propidium iodide in PBS at room temperature for 1 h. Stained cells then analyzed by Cell Lab Quanta SC flow cytometry (Beckman Coulter, Brea, CA, USA).

### Annexin V/PI double-staining assay

In order to analyze the apoptosis status of cells after indicated treatment, cells were stained by Alexa flour 488 annexin V/Dead cell apoptosis kit (Invitrogen, Carlsbad, CA, USA) according to manufacturer’s protocol. Stained cells were analyzed by Cell Lab Quanta SC flow cytometry (Beckman Coulter, Brea, CA, USA).

### Whole-genome sequencing

Human lung adenocarcinoma epithelial cell line A549 was sequenced for their whole genomes. Genomic DNA materials were extracted by using QIAamp DNA Mini kit (QIAGEN, Germantown, MD, USA.) according to manufacturer’s protocol. DNA degradation and contamination were monitored on 1% agarose gels and DNA concentration was measured using Qubit^®^ DNA Assay Kit in Qubit^®^ 2.0 Flurometer (Life Technologies, CA, USA). A total amount of 1.0 μg DNA per sample was used as input material for the DNA sample preparations. Sequencing libraries were generated using NEBNext^®^ DNA Library Prep Kit following manufacturer’s recommendations and indices were added to each sample. The genomic DNA was randomly fragmented to a size of 350 bp by shearing, then DNA fragments were end polished, A-tailed, and ligated with the NEBNext adapter for Illumina sequencing, and further PCR enriched by P5 and indexed P7 oligos. The PCR products were purified (AMPure XP system) and resulted libraries were analyzed for size distribution by Agilent 2100 Bioanalyzer and quantified using real-time PCR.

### Statistics

All samples were used for statistical analysis. The investigator was aware of the sample allocation during the experiment and when assessing its outcome for all animal experiments. For all experiments, at least three independent biological replicates of each conditions were analyzed. Estimated variation within each experiment group is similar. The difference between two groups was analyzed by two-tailed unpaired Student’s *t* test. The *p* value, which is <0.05, was considered as statistically significant. Center value is defined as mean value, and sem is used to calculate and plot error bars from raw data.

## Results

### USP24 positively regulates drug resistance during cancer therapy

**Fig. 1:**
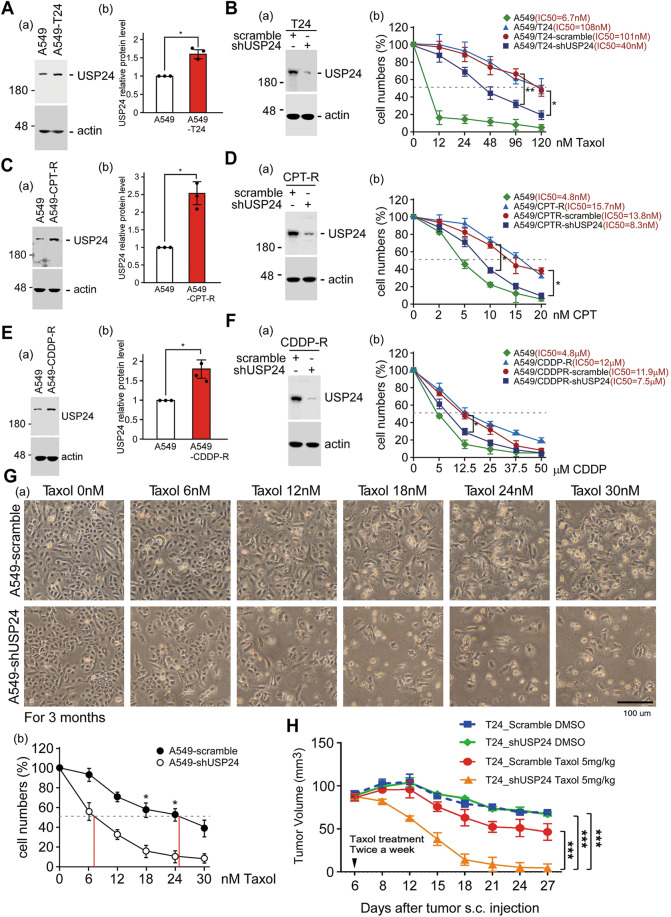
USP24 positively regulates drug resistance. The levels of USP24 in A549 cells and the drug-resistant cell lines A549-T24, A549-CPT-R, and A549-CDDP-R cells were determined by Western blotting (**A**, **C**, **E**). The cytotoxic effects of Taxol, CPT, and CDDP in normal and drug-resistant A549 cells with or without USP24 knockdown were studied (**B**, **D**, **F**). A549 cells with or without USP24 knockdown were treated with Taxol for three months, and the IC_50_ of Taxol in the cells was determined (**G**). T24 cells with or without USP24 knockdown were injected into NOD/SCID mice, and the mice were subsequently injected with Taxol at the indicated times. Tumor volume was determined (**H**). The results from three independent experiments were statistically analyzed using a *t*-test: *p < 0.05, **p < 0.01, ***p < 0.005.

Our previous studies indicated that USP24 is upregulated in cancer cells and that tumor-associated macrophages to promote lung cancer malignancy [13]. Herein, USP24 was knocked down in both cancer cells and macrophages to study the effect of USP24 on cancer malignancy (Supplementary Fig. 1). The loss of USP24 in cancer cells and macrophages significantly inhibited the malignant ability of cancer cells, which indicates that USP24 is a potential target for the development of drugs. Because drug resistance is a major factor to trigger the recurrence and malignancy of cancer [22], the role of USP24 in drug resistance during therapy needs to be clarified (Fig. 1). The chemotherapy drugs Taxol, camptothecin (CPT), and cisplatin were used to induce drug resistance in cell lines, and the level of USP24 was found to be increased in these resistant cell lines (Fig. 1A, C, E). Knockdown of USP24 in these drug-resistant cell lines partially repressed the drug resistance (Fig. 1B, D, F). The other cancer cell lines, Hone-1 and HCT116 cells, were also used to study the role of USP24 in CPT- and oxaliplatin-induced drug resistance (Supplementary Figs. 2A, B and 3A, B). The loss of USP24 partially reversed the cytotoxicity of CPT and oxaliplatin in Hone-1R and HCT116R cancer cells, respectively. In addition, Taxol-sensitive A549 cells with or without USP24 were treated with Taxol for 3 months to generate drug-resistant cell lines (Fig. 1G). Interestingly, Taxol treatment for three months induced drug resistance in the A549 cell line (IC50 = 25 nM), but the loss of USP24 completely blocked drug resistance (IC50 = 6.6 nM). Taxol-induced A549 drug-resistant cell line, T24, with or without USP24 knockdown were injected into SCID mice, and then the mice were treated with Taxol (Fig. 1H). The loss of USP24 significantly enhanced the cytotoxic effect of Taxol to reduce tumor size and tumor weight (Fig. 1H). Taken together, it would be better to use the cocktail treatment, USP24 inhibitor, and chemotherapeutic drugs, in the cancer patients before the drug resistance has emerged.

### USP24 stabilizes ABC transporters to pump out a drug from cancer cells

**Fig. 2:**
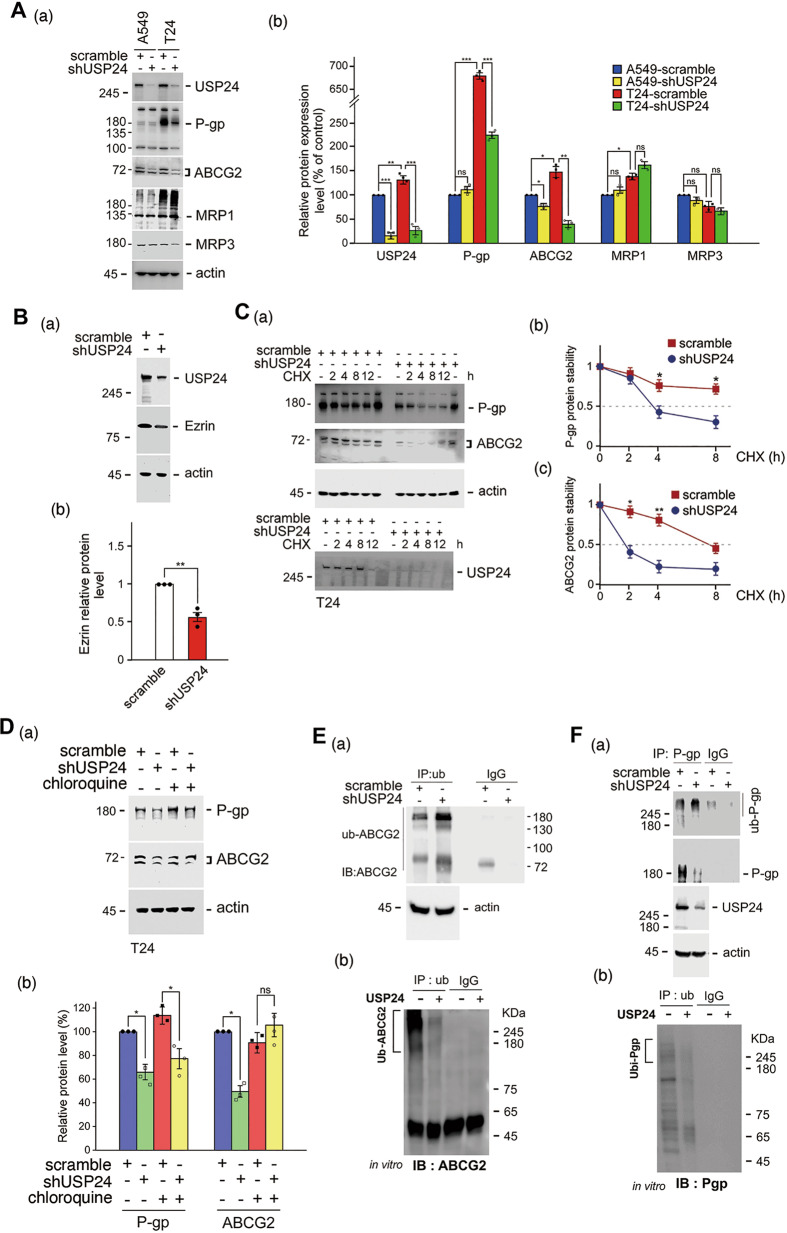
USP24 stabilizes ABC transporters, which pump out drugs. The levels of P-gp, ABCG2, MRP1, MRP3, and actin in A549 cells and Taxol-resistant A549, T24 cells, were studied by Western blotting with various antibodies (**A**). The level of ezrin in A549 cells with or without USP24 knockdown was determined by Western blot analysis (**B**). P-gp and ABCG2 protein stability in T24 cells with or without cycloheximide or chloroquine treatment and USP24 knockdown was studied by Western blot analysis with anti-P-gp and anti-ABCG2 antibodies (**C**, **D**). The levels of P-gp and ABCG2 ubiquitination in A549 cells with or without USP24 knockdown were studied by Western blot analysis (**E**(a), **F**(a)). In in vitro enzyme assays were performed. IP with anti-Ubiquitin collected ubiquitinated proteins as substrates to react with purified recombinant USP24, and the levels of ubi-ABCG2 and ubi-P-gp were then studied by IB with anti-ABCG2 and anti-P-gp antibodies (**E**(b), **F**(b)). The results from three independent experiments were statistically analyzed using a *t*-test: *p < 0.05, **p < 0.01, ***p < 0.005.

**Fig. 3:**
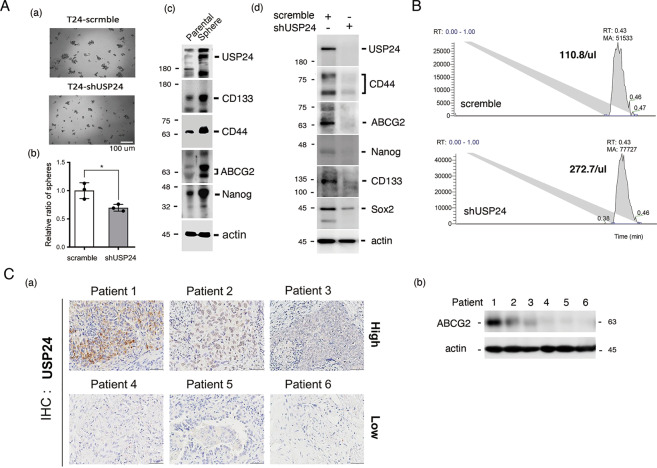
USP24 promotes cancer stemness characteristics. The ability of T24 cells with or without USP24 knockdown to form spheres was studied (**A**(a) and (b)). The levels of various cancer stemness markers in parental and sphere cells (**A**(c)) and in T24 cells with or without USP24 (**A**(d)) were studied by IB with the indicated antibodies. The Taxol concentration inside cells with or without USP24 knockdown was determined by LC/MS/MS (**B**). The clinical correlation between the levels of USP24 and ABCG2 was studied by IHC and IB with anti-USP24 and anti-ABCG2 antibodies (**C**). The results from three independent experiments were statistically analyzed using a *t*-test: *p < 0.05.

Since ABC transporters are critical for drug resistance [19], the levels of P-gp, ABCG2, and MRP1 were studied and found to be increased in Taxol-induced T24 lung cancer cells compared with A549 cells or other resistant cell lines. After USP24 knockdown, the levels of P-gp, ABCG2, and ezrin were decreased, which indicated that USP24 as a deubiquitinating enzyme may stabilize P-gp, ABCG2, and ezrin to enhance the ability of pumping out of a drug from cancer cells (Fig. 2A, B and Supplementary Figs. 2C, D and 3C, E). Furthermore, knockdown of USP24 decreased the protein stability of P-gp and ABCG2 (Fig. 2C). This effect of USP24 in P-gp and ABCG2 could be reversed by MG132 and chloroquine treatment, respectively (Fig. 2D and Supplementary Fig. 4A, B), which suggested that USP24 stabilizes P-gp and ABCG2 in a proteasome- and lysosome-dependent manner, respectively [30]. Moreover, USP24 also stabilized ezrin from proteasomal degradation (Supplementary Fig. 4C). USP24 could interact with P-gp, ABCG2, and ezrin, and the knockdown of USP24 increased the ubiquitination of P-gp and ABCG2 (Fig. 2E(a), F(a) and Supplementary Fig. 5A, C). The in vitro enzyme assay showed that the purified recombinant USP24 could decrease ubiquitinated signal of ABCG2 and P-gp (Fig. 2E(b), F(b)). These findings indicated that USP24 stabilizes these ABC transporters, which pump out drugs (Fig. 2E, F and Supplementary Fig. 5). According to previous studies, ezrin can assist ABC transporters in the cell membrane [21], herein, we found not only that USP24 could stabilize ezrin but also that ezrin could increase ABCG2 (Fig. 2B and Supplementary Fig. 5D). Previous studies have also shown that cancer stemness is related to drug resistance [20]. The knockdown of USP24 decreased the levels of CD44 and sphere formation (Fig. 3A(a, b) and Supplementary Fig. 5E). In addition, the levels of USP24, CD133, CD44, ABCG2, and Nanog in spheroid cells were higher than their levels in the parental cells (Fig. 3A(c)). The loss of USP24 significantly decreased the levels of cancer stemness markers, including CD44, ABCG2, Nanog, CD133, and Sox2 (Fig. 3A(d)). We then directly detected the concentration of Taxol inside the cells with or without USP24 knockdown by mass spectrometry (Fig. 3B). The loss of USP24 increased the levels of Taxol from 110.8 to 272.7 nM inside the drug-resistant T24 cells treated with 1000 nM Taxol (Fig. 3B and Supplementary Fig. 6). The relationship among USP24, ABCG2, and cancer stemness markers was then studied in clinical lung cancer cohorts (Fig. 3C). The data indicated a highly positive correlation between the levels of USP24 and ABCG2 in patients with lung cancer (Fig. 3C). All the results show that USP24 stabilizes ABC transporters and increases cancer stemness characteristics to induce drug resistance.

### USP24 enters the nucleus to induce genomic instability and decrease DNA damage repair activity under DNA damage conditions

**Fig. 4:**
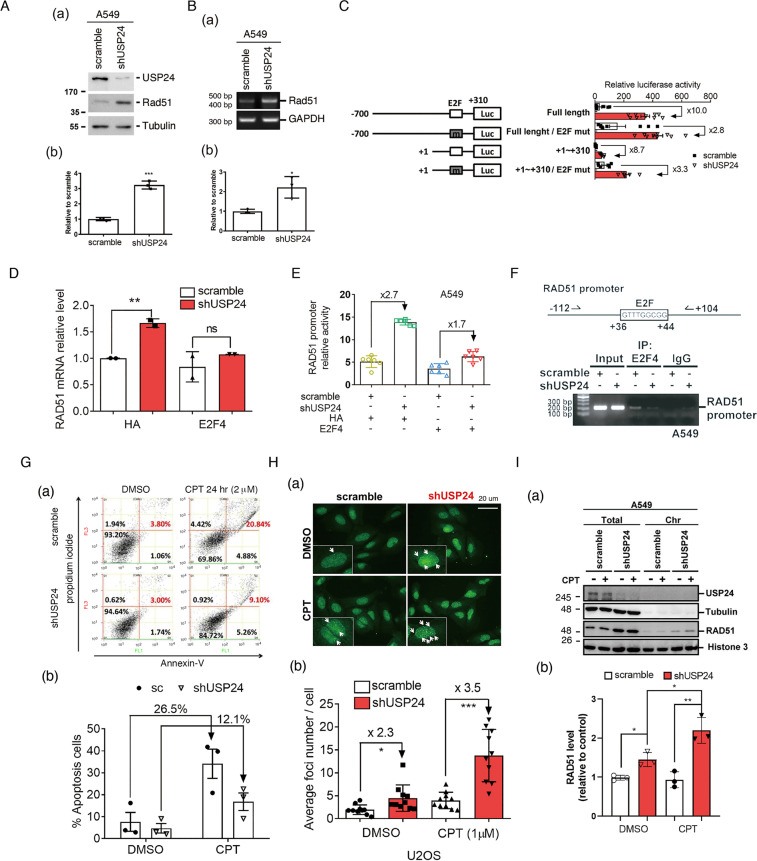
USP24-stabilized E2F4 represses Rad51 transcriptional activity. The protein (**A**) and mRNA (**B**) levels of Rad51 in A549 cells with or without USP24 knockdown were studied by Western blot and RT-PCR assays. The luciferase activity driven by the promoter of *rad51* with or without deletion or mutation of the E2F-binding site was studied (**C**). The mRNA level of Rad51 (**D**) and luciferase activity driven by promoter of *rad51* (**E**) with or without USP24 in the absence or presence of E2F4 overexpression was studied. The recruitment of E2F4 to the promoter of *rad51* in A549 cells in the absence or presence of USP24 was studied by ChIP assay (**F**). The cell viability and DDR activity of A549 cells with or without CPT treatment and USP24 knockdown were studied by flow cytometry (**G**) and Rad51 foci assays (**H**). The distribution of Rad51 colocalized with chromatin in cells with or without USP24 knockdown under CPT treatment was studied by Western blotting analysis with the indicated antibodies (**I**). The results from three independent experiments were statistically analyzed using a *t*-test: *p < 0.05, **p < 0.01, ***p < 0.005.

What is the reason(s) of why the effect of USP24 on the drug resistance between drug-sensitive and drug-resistant cancer cell lines is distinguishable? Our RNA-sequencing data indicated that the mRNA level of Rad51 was dramatically increased by USP24 knockdown (data not shown). Indeed, the protein and mRNA levels of Rad51 were increased in USP24 knockdown A549 cells (Fig. 4A, B). To study the transcriptional activity of Rad51, we found that the E2F-binding site in the promoter of *rad51* is critical for USP24-mediated Rad51 expression (Fig. 4C and Supplementary Fig. 7). Previously, we have known that E2F4 is the substrate of USP24 [31], and herein we found that overexpression of E2F4 reversed the effect of USP24-KD on the mRNA level and promoter activity of Rad51, which suggested that USP24-stabilized E2F4 might repress Rad51 expression (Fig. 4D, E). We then found that E2F4 could be recruited within the promoter of *rad51* and that the knockdown of USP24 could abolish this recruitment (Fig. 4F). Therefore, we studied the role of USP24 in DDR activity during cancer progression (Fig. 4G, H). First, the knockdown of USP24 inhibited the CPT-induced apoptosis, cytotoxicity, and sub-G1 population in A549 cells (Fig. 4G and Supplementary Fig. 8), but this effect was reversed by E2F4 overexpression or Rad51 knockdown (Supplementary Fig. 9). To study the role of USP24 in DDR, we found that the knockdown of USP24 increased the foci number and recruitment of Rad51 into chromosomes (Fig. 4H, I). Together, USP24 decreased DDR activity during cancer progression to be beneficial for chemotherapy-induced cell death but might induce genomic instability and ultimately leads to drug resistance.

**Fig. 5:**
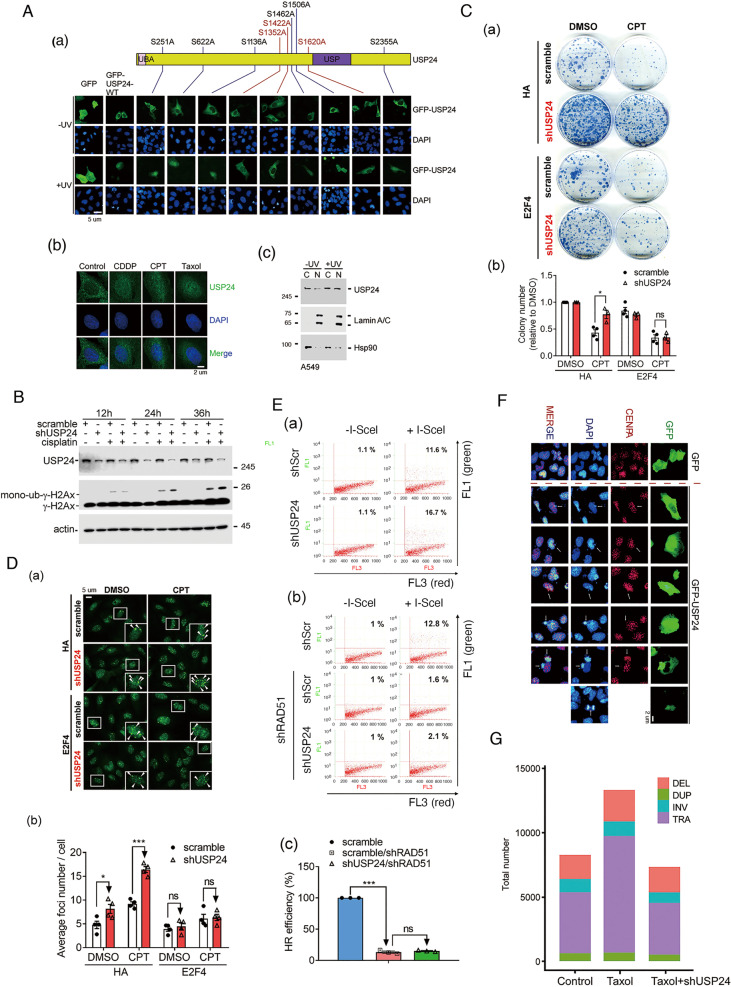
USP24 enters the nucleus to induce genomic instability. The localization of GFP, GFP-USP24 and various mutants of GFP-USP24 under normal or UV exposure conditions was studied by IF (**A**(a)). The level of USP24 in the cytoplasm and nuclei of A549 cells treated with CDDP, CPT, and Taxol (**A**(b)) with or without UV exposure (**A**(c)) was studied by IF and IB with the indicated antibodies. The levels of γ-H2AX and monoubiquitinated γ-H2AX in A549 cells with or without USP24 knockdown and cisplatin treatment were studied by Western blot analysis with the indicated antibodies (**B**). The viability of A549 cells treated with CPT, with or without USP24 knockdown and E2F4 overexpression was studied by colony-formation assay (**C**). The Rad51 foci was studied by anti-Rad51 antibodies in A549 cells with or without E2F4 overexpression and USP24 knockdown (**D**). HR-mediated DDR was studied by flow cytometry assay in A549 cells with or without knockdown of Rad51 and USP24 (**E**). The localization of GFP, GFP-USP24, CENPA, and DAPI was studied by IF with the indicated antibodies (**F**). The whole genomes of A549, T24, and USP24-knockdown T24 cells were sequenced by WGS and analyzed using Circos software to study the structure variants (SVs) (**G**). The results from three independent experiments were statistically analyzed using a *t*-test: *p < 0.05, ***p < 0.005.

Next, we further investigate the mechanism how does USP24 modulate DDR. Herein, we found that USP24 could be transported into the nucleus in lung cancer cells under UV exposure, CPT, and cisplatin treatment (Fig. 5A(b, c) and Supplementary Fig. 10). All the conserved ATM phosphorylation conserve sequences within USP24 were individually mutated and then overexpressed in cells, and the distributions of the USP24 mutant proteins following UV exposure were then determined (Fig. 5A(a)). Three sites, S1352, S1422, and S1620, are important for the nuclear localization of USP24. The knockdown of USP24 decreased the cytotoxicity of CPT (Fig. 5C), increased the γ-H2Ax and mono-ub-γ-H2Ax levels (Fig. 5B), increased the foci number (Fig. 5D), and increased the HR-mediated GFP signal (Fig. 5E), but E2F4 abolished all of the effects. Furthermore, the overexpression of GFP-USP24 caused anaphase bridge and ultrafine bridge formation (Fig. 5F), which implies that USP24 induces genomic instability and thereby increases the tumor mutation burden (TMB). The whole genomes of A549 and Taxol-induced drug-resistant cell line, T24, with or without USP24 knockdown were sequenced by whole-genome sequencing, and the results were analyzed using Circos software (Fig. 5G and Supplementary Fig. 11). The findings revealed that Taxol-induced drug resistance increased the proportions of structure variants and translocations, but no difference in deletions, duplications, or inversions, and these effects were abolished by the knockdown of USP24, which indicates that USP24 promotes drug resistance not only by increasing the pumping out of a drug from cells but also by inducing genomic instability to enhance the TMB (Fig. 5G). In summary, USP24 enters the nucleus to induce genomic instability by inhibiting DDR, which might be the reason of how to distinguish the effect of USP24 on drug resistance between drug-sensitive and drug-resistant cell lines.

### Novel-specific USP24 inhibitors block the enzyme activity of USP24 to inhibit the drug resistance during cancer therapy

**Fig. 6:**
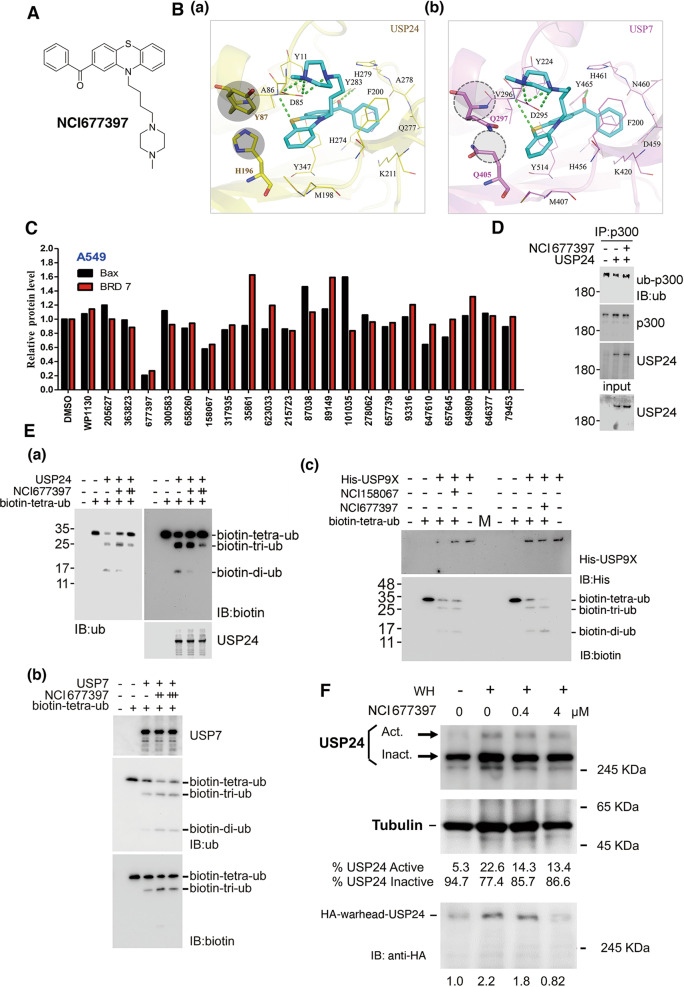
Docking pose of NCI677397. The NCI677397 molecule (**A**). NCI677397 (blue) is docked into USP24 (**B**(a)) and USP7 (**B**(b)). The different residues are highlighted by a gray circle. The dashed green lines denote hydrogen bonds. The binding sites of USP24 and USP7 are shown in yellow and pink, respectively. The binding site residues are labeled and shown as lines. The levels of the USP24 substrates, Bax and BRD7 in A549 cells treated with various candidate USP24 inhibitors were determined by IB with anti-Bax and anti-BRD7 antibodies (**C**). The ubiquitination of p300 by IP with p300 antibodies in A549 cells with or without USP24 purified recombinant protein or USP24 inhibitor treatment was studied by Western blot analysis with anti-ubiquitin antibodies (**D**). The enzyme activity of purified recombinant USP24 (a), USP7 (b), and USP9X (c) proteins in the absence or presence of NCI677397 or NCI158067 was studied with an in vitro enzyme assay using biotin-tetra-ubiquitin as the substrate (**E**). The effect of NCI677397 was studied by an in vitro enzyme assay with or without NCI677397 in the presence of an HA-warhead (WH). Active and inactive USP24 were studied by IB with anti-USP24 and anti-HA antibodies (**F**).

Our findings suggested that screening of a novel-specific USP24 inhibitor to prevent drug resistance during cancer therapy. Due to the unavailability of a crystal structure for USP24, a homology model was created using the MODELLER [27] module within the Chimaera [28] software. The USP7 (PDB: 5N9R) protein structure was used as a template. The protein structures were prepared for docking using the LeadIT software [29]. A docking radius of 10 Å from the co-crystal ligand of USP7 was used for the binding site. The binding site of the USP24 homology model was determined by alignment to the USP7 protein structure. The compound NCI677397 was prepared by protonation in aqueous solution. Molecular docking was performed using the LeadIT and generated poses for analysis. The docking strategy used a hybrid enthalpy/entropy approach with default scoring parameters. We used the structure-based strategy to screen inhibitors from the National Cancer Institute (NCI) compound library [32] (Fig. 6A and Supplementary Fig. 12). To further elucidate the interactions between NCI677397 and USP24, the docking analysis showed that a number of hydrogen bonds between NCI677397 and the binding site in USP24 (Fig. 6B(a)). The 1‐methylpiperazine structure contains two nitrogen atoms that facilitate the binding of three hydrogen bonds to residue D85. Two additional hydrogen bonds form between NCI677397 and residues A86 and Y283. Residue A86 donates a hydrogen to a sulfur on the phenothiazine ring, and a carbonyl oxygen functions as a hydrogen acceptor for residue Y283. The aromatic rings of NCI677397 form hydrophobic interactions with nonpolar residues such as A86, Y87, F200, Y283, and Y347. NCI677397 was also docked into USP7 to compare the interactions observed with the USP24 model (Fig. 6B(b)). Similar to the results obtained with USP24, NCI677397 formed five hydrogen bonds with USP7. Further analysis revealed further substitutions between the binding sites in USP24 and USP7 (Fig. 6B(a, b)). Two USP24 residues, Y87 and H196, are replaced by two glutamines in USP7. The USP24 residues Y87 and H196 contain bulky side chains that can restrict the size of the binding site. In addition, these USP24 residue side chains consist of aromatic rings, which form additional hydrophobic interactions with NCI677397. In contrast, USP7 contains residues Q297 and Q405, and the positioning of Q297 and Q405 does not facilitate hydrophobic interactions with the phenothiazine ring of NCI677397. As a result, NCI677397 forms weaker hydrophobic interactions within the binding site of USP7 compared with those found with USP24. The inhibitory effects of several compounds harvested from the compound library on the catalytic activity of USP24 were studied using the substrates Bax and BRD7 (Fig. 6C). Two compounds, NCI677397 and NCI158067, decreased the levels of Bax, BRD7, and p300 (Fig. 6C and Supplementary Fig. 13). The compound WP1130, which can inhibit lymphoma progression [33], did not inhibit USP24 activity with these substrates in lung cancer (Fig. 6C). The treatment of A549 cells with NCI677397 also increased the ubiquitination of p300 by NCI677397 (Fig. 6D). These two compounds also inhibited the catalytic activity of recombinant purified USP24 protein (Fig. 6E(a) and Supplementary Fig. 14A). Interestingly, NCI677397 did not block the catalytic activity of USP7, USP9x, or USP10, but NCI158067 inhibited that of USP7 and USP10, which indicates that NCI677397 exhibits higher specificity in blocking USP24 enzyme activity (Fig. 6E(b, c) and Supplementary Fig. 14B, C). We also used a ubiquitin suicide probe (Warheads) to study the inhibitory effect of NCI677397 (Fig. 6F), and the data indicated that NCI677397 can inhibit the activity of USP24. All the data indicate that NCI677397 contains higher specificity and efficacy on the inhibition of USP24.

### NCI677397 inhibits chemotherapy-induced drug resistance in vitro and in vivo

**Fig. 7:**
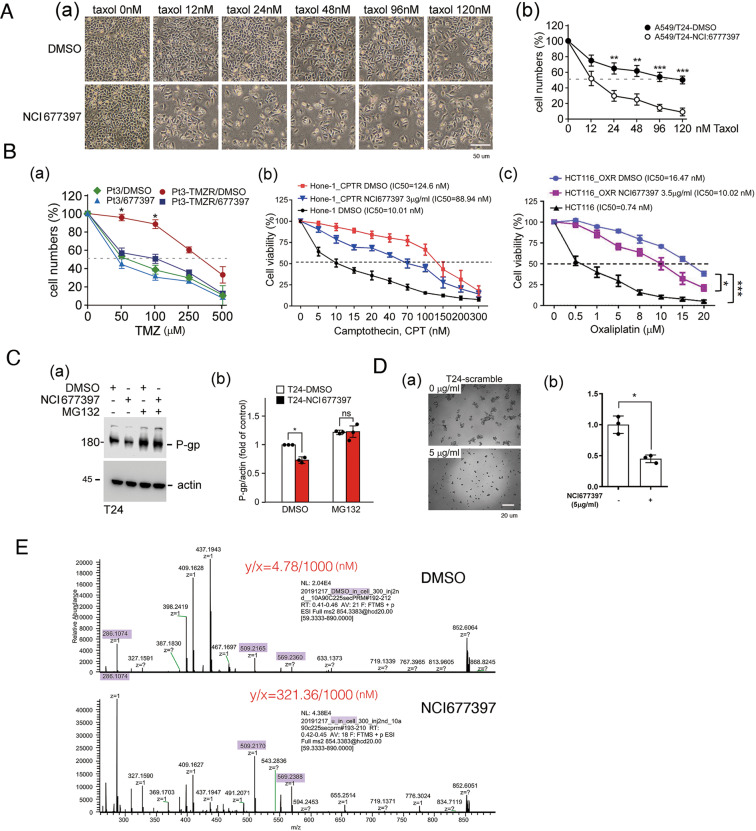
NCI677397 inhibits chemotherapy-induced drug resistance in vitro. The cytotoxicity of Taxol to Taxol-resistant A549 lung cancer cells, T24 cells (**A**), TMZ to TMZ-resistant GBM cells from patient 3 (Pt’3 TMZR) (**B**(a)), CPT to CPT-resistant Hone-1 cells (**B**(b)), and oxaliplatin to oxaliplatin-resistant HCT116 cells (**B**(c)) in the presence or absence of NCI677397 treatment were determined. The P-gp protein level in T24 cells treated with a USP24 inhibitor was studied by Western blot analysis with the indicated antibodies (**C**). The ability of T24 cells with or without NCI677397 treatment to form spheres was determined (**D**). The concentration of Taxol inside T24 cells treated with a USP24 inhibitor was determined by LC/MS/MS (**E**). The results from three independent experiments were statistically analyzed using a *t*-test: *p < 0.05, **p < 0.01, ***p < 0.005.

**Fig. 8:**
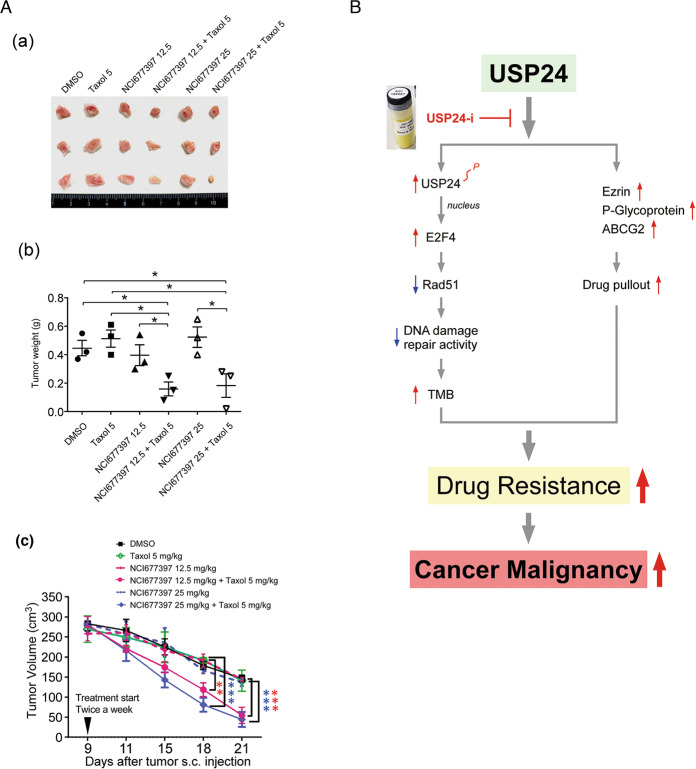
NCI677397 inhibits chemotherapy-induced drug resistance in vivo. The T24 cells were injected into SCID mice, and subsequently were treated with Taxol and a USP24 inhibitor at the indicated times. The tumor nodules (**A**(a)), tumor weight (**A**(b)), and tumor volume (**A**(c)) were determined. The results from three independent experiments were statistically analyzed using a *t*-test: *p < 0.05, **p < 0.01, ***p < 0.005. The working model, USP24 promotes drug resistance by decreasing genomic stability and increasing the pumping out of drugs, was shown (**B**).

Therefore, we studied the inhibitory effect of NCI677397 on drug resistance in the different cancer cell lines (Fig. 7 and Supplementary Fig. 15). NCI677397 significantly blocked the induction of drug resistance in lung cancer, T24, by Taxol (Fig. 7A), GBM, Pt3-TMZR, and U87-R, by temozolomide, NPC, Hone-1-CPTR, by CPT, and colorectal cancer (CRC), HCT116-OXR, by oxaliplatin (Fig. 7B and Supplementary Fig. 15). In addition, NCI677397 treatment decreased the level of P-gp (Fig. 7C), and inhibited the spheroid formation ability of T24 cells (Fig. 7D). This finding suggests that the loss of USP24 catalytic activity reduces cancer stemness characteristics, which might be important for drug resistance [20]. Subsequently, NCI677397 increased the concentration of Taxol from 4.78 to 321.36 nM inside cells under 1000 nM Taxol treatment (Fig. 7E). The effect of NCI677397 in inhibiting drug resistance was also addressed in vivo (Fig. 8A). The cytotoxic effect of Taxol on inhibiting the tumor size and weight in SCID mice was enhanced by NCI677397 cotreatment (Fig. 8A), but the body weight was not affected (Supplementary Fig. 16). A highly positive correlation between USP24 and cancer stemness markers was also found in cancer cell-injected SCID mice (Supplementary Fig. 17). Finally, because USP24 promotes lung cancer malignancy, we also studied the effects of NCI677397, NCI158067, and other compounds from the NCI library in blocking lung cancer migration (Supplementary Figs. 18 and 19), and the data indicated that both NCI677397 and NCI158067 could inhibit lung cancer migration, but the other compounds not.

## Discussion

USP24 increases the TMB and induces the pumping of drugs out of cells to induce drug resistance. A novel, USP24-specific inhibitor, NCI677397, could stabilize the genome, reduce cancer stemness characteristics, and increase the concentration of a drug inside cells to block the emergence of drug resistance (Fig. 8B).

The ABC transporter belongs to a large and ubiquitous superfamily of ATP-dependent transporters that participate in a wide range of physiological and pathological processes, including the transportation of many cargoes in and out of cells, particularly large molecules such as drugs [19]. The chemotherapeutic treatments for cancers often induce mutations in pleotropic drug resistance-related proteins and MRPs [1–3]. Because ABC transporters are involved in the emergence of drug resistance in various cancer types treated with various drugs, understanding the mechanisms regulating the activity of ABC transporters during cancer treatment is critical for inhibiting drug resistance. In this study, we found that USP24 can stabilize P-gp, ABCG2, and ezrin in a 26S proteosome- or lysosome-dependent manner, which leads to the pumping out of the drug from cells and ultimately resulting in drug resistance [30]. According to previous studies, the ERM proteins act as linkers between the plasma membrane and actin skeleton to transport ABCG2 from the cytoplasm to the plasma membrane and thus activate the activity of ABC transporters [21]. In this study, we not only found that USP24 can stabilize ezrin but also discovered that ezrin can increase the level of ABCG2. Most previous studies have indicated that the regulation of transcriptional activity contributes to the upregulation of ABC transporters during cancer therapy [19]. Few studies on the protein stability of ABC transporters have been reported. A previous study indicated that the DUB PSMD4 promotes tumor growth and chemoresistance by stabilizing the ALK2 receptor during the initiation of the BMP6 signaling pathway [34]. Herein, we provide direct evidence showing that P-gp, ABCG2, and ezrin are substrates of USP24. Furthermore, we also screened the specific novel USP24 inhibitor NCI677397 and found that this inhibitor blocks drug resistance by inhibiting ABC transporters. NCI677397 treatment not only decreased the levels of ABC transporters but also increased the concentration of Taxol inside cancer cells.

In addition to the ABC transporters, the genomic instability-induced TMB is another major inducer of drug resistance under various cancer therapeutic strategies [22]. Our previous studies indicated that an increase in USP24 at the late stages of cancer progression promotes cancer malignancy [12, 13]. Herein, we found that USP24 decreased HR-mediated DDR activity and thus induces genomic instability. The loss of USP24 by shRNA knockdown or treatment with the USP24 inhibitor NCI677397 inhibited drug resistance under Taxol treatment. In this study, we found that USP24-stabilized E2F4 repressed Rad51 to decrease HR-mediated DDR activity and subsequently induced genomic instability. In addition, the knockdown of USP24 increased the colony number and decreased the levels of sub-G1 population in CPT-treated cancer cells, which suggests that USP24 increases the cytotoxicity of CPT and indicates that USP24 inhibits the DDR. Under continuous DNA damage induced by CPT treatment, γ-H2AX, which is a marker of DDR activity, was recruited to foci. However, with short-term DNA damage by UV exposure, γ-H2AX is a marker of DNA damage during the recovery period (Supplementary Fig. 20). Several DUBs have been reported to be related to the regulation of DDR activity [35]. USP14 regulates the DDR by targeting RNF168-dependent ubiquitination [36]. In addition, USP51 deubiquitylates H2AK13 and 15ub and regulates the DNA damage response [37]. Recent studies also found a distinct DUB class, ZUFSP, which is related to genomic stability through recruitment to DNA lesions [38]. USP5 regulates CRC cell growth by stabilizing the translation elongation factor Tu, which is related to DDR [39]. Taken together, the results show that DUBs are involved in DDR activity and might be potential targets for future drug development. Our evidences show that only USP24 regulates ABC transporters and DDR activity simultaneously, and we also found a novel USP24 inhibitor, NCI677397, that can inhibit drug resistance by inhibiting the pumping out of a drug from cancer cells as well as maintaining the genomic stability to inhibit the cancer stemness characteristics.

Several USP inhibitors have been developed to inhibit cancer progression [6]. For example, the USP7 inhibitors, GEN6776, FT671, and FT827 can repress cancer growth and metastasis [40]. In this study, we screened a novel-specific USP24 inhibitor, NCI677397, and found that this inhibitor can inhibit ABC transporters and genomic instability and thereby prevents drug resistance to chemotherapy in different cancer types. Interestingly, according to this study, the US24 inhibitor NCI677397 only partially reverses the cytotoxicity of Taxol through increasing ABC transporter degradation in drug-resistant cell lines but completely abolishes drug resistance in drug-sensitive cancer cell lines by inhibiting ABC transporters and genomic instability. Therefore, patients with cancer should be treated with chemotherapeutic drugs and USP24 inhibitors together prior to the emergence of drug resistance. Why did we select USP7 as the parent template to model the USP24 structure? First, we used the software program to study the similarity between USP24 and all other USPs. The data indicated that the catalytic domain of USP24 exhibited the highest similarity to that of USP7. Second, previous studies showed that USP7 and USP24 possess the same substrate, such as p53, which indicates a higher similarity between these structures [41, 42].

In the future, the NCI677397 will be modified to optimize the inhibition of drug resistance under chemotherapy, and the safety of this drug will also be assessed in vitro and in vivo. In addition, because genomic instability and the pumping out of drugs are critical and general issues in the induction of drug resistance, we will study the effect of USP24 inhibitors on drug resistance induced by targeted therapy and immune therapy in the future.

## Supplementary information

Supplementary material

Supplementary Fig.1

Supplementary Fig.2

Supplementary Fig.3

Supplementary Fig.4

Supplementary Fig.5

Supplementary Fig.6

Supplementary Fig.7

Supplementary Fig.8

Supplementary Fig.9

Supplementary Fig.10

Supplementary Fig.11

Supplementary Fig.12

Supplementary Fig.13

Supplementary Fig.14

Supplementary Fig.15

Supplementary Fig.16

Supplementary Fig.17

Supplementary Fig.18

Supplementary Fig.19

Supplementary Fig.20
