# Supplementary material for: USP24 promotes drug resistance during cancer therapy
Source: Cell Death Differ. 2021 Apr 12;28(9):2690–707. doi: 10.1038/s41418-021-00778-z (PMC8408266; doi:10.1038/s41418-021-00778-z)
Supplement: Supplementary file 1 — Supplementary material [file 41418_2021_778_MOESM1_ESM.docx]

**Supplementary Legends**

**Suppl.Fig.1.** USP24 in lung cancer cells and macrophages promotes lung cancer malignancy. The migratory ability of lung cancer cells with or without USP24 knockdown, and also treated with conditional medium collected from macrophages with or without USP24 knockdown was studied by migration assay (A), and the quantitation of the migratory lung cancer cells was performed after three independent experiments (B). The levels of USP24 and actin were studied in A549 cells by WB with indicated antibodies (C). The results were subjected to statistical analysis by t-test, p***<0.005, after three independent experiments.

**Suppl.Fig.2.** The effect of USP24 on HONE1-CPTR cells. The cytotoxicity of various concentration of CPT in HONE1 and HONE1-CPTR cells with or without USP24 knockdown was studied (A), and was quantified after three independent experiments (B). The levels of USP24, P-gp, ABCG2, MRP1 and MRP3 were studied in the HONE1 and HONE1-CPTR cells with or without USP24 knockdown by Western blot with the indicated antibodies (C and D). The results were subjected to statistical analysis by t-test, p* < 0.05, after three independent experiments.

**Suppl.Fig.3.** The effect of USP24 on HCT116-OX5R cells. The IC50 of oxaliplatin in HCC116 and oxaliplatin resistant CRC cell line, HCT116-OX5, were determined (A, B). The levels of USP24, P-gp and ABCG2 in HCT116 and HCT116-OX5 were studied by IB with indicated antibodies (C). The cytotoxicity of oxaliplatin in HCT116 with or without USP24 knockdown was studied (D), and the levels of P-gp and ABCG2 in HCT116-OX5 with or without USP24 knockdown were studied by IB with indicated antibodies (E). The results were subjected to statistical analysis by t-test, p* < 0.05, p*** < 0.005, after three independent experiments.

**Suppl.Fig.4**. The effect of MG132 on the stability of P-gp, ABCG2 or ezrin under USP24 knockdown in A549 or T24 cells. The levels of P-gp (A), ABCG2 (B) and ezrin (C) in A549 and T24 cells in the present of MG132 with or without USP24 knockdown was studied by Western blotting analysis with the indicated antibodies, and were quantified after three independent experiments. The results were subjected to statistical analysis by t-test, p* < 0.05, p** < 0.01, after three independent experiments.

**Suppl.Fig.5.** Ezrin positively regulates ABCG2. The interaction between USP24 with P-gp (A), ABCG2 (B) and Ezrin (C) by IB with the indicated antibodies. The level of ABCG2 in A549 cells with or without Flag-ezrin overexpression was studied by Western blotting analysis (D). The levels of CD44 in T24 cells with or without USP24 knockdown were studied by Western blot analysis with the indicated antibodies (E).

**Suppl.Fig.6**. The detail information of the LC/MS/MS in detecting the dose of taxol inside A549-T24 drug resistance cells with or without knockdown of USP24 was show here.

**Suppl**.Fig.7. The E2F binding site and E2F-like binding motif within promoter of Rad51 was show here (A). The luciferase activity driven by various truncated promoters of Rad51 were studied in A549 cells (B and C)

**Suppl.Fig.8.** The cytotoxicity of CPT was studied in A549 cells with or without USP24 knockdown by colony formation assay (A) and flow cytometry assay (B). The results were subjected to statistical analysis by t-test, p* < 0.05 and p** < 0.01, after three independent experiments.

**Suppl.Fig.9.** The sub-G1 in A549 cells with or without USP24 knockdown or E2F4 overexpression was studied by Flow cytometry (A). The sub-G1 in A549 cells with or without USP24 knockdown or Rad51 knockdown under CPT exposure was studied by Flow cytometry assay (B). The results were subjected to statistical analysis by t-test, p** < 0.01, after three independent experiments.

**Suppl.Fig.10.** The signal and localization of GFP-USP24 and γ-H2AX in A549 cells with or without UV exposure were studied by IF with the indicated antibodies.

**Suppl.**Fig.11. A549 or T24 cells with or without USP24 knockdown were treated with Taxol, and then the genomes were extracted for whole genome sequencing (WGS). The genome sequences were analyzed with Circos software.

**Suppl.Fig.12.** The structural interaction between USP24/USP7 and the compounds, NCI677397 and NCI158067.

**Supp.Fig.13.** The levels of p300 and BRD7 in A549 cells treated with NCI677397 treatment were studied by Western blotting analysis with the indicated antibodies. The results were subjected to statistical analysis by t-test, p* < 0.05, p** < 0.01, after three independent experiments.

**Suppl.Fig.14.** *in vitro* enzyme assay. The effect of NCI158067 in USP24 *in vitro* enzyme assay was studied (A). The effects of NCI158067 (B) in USP7 *in vitro* enzyme assay were studied. The effects of NCI677397 and NCI158067 in USP10 *in vitro* enzyme assay were studied (C).

**Suppl.Fig.15.** The cytotoxicity of TMZ in GBM Pt’3 and Pt’3-R cells(A) or U87 and U87R (B) treated with NCI677397 treatment was studied, and were quantified after three independent experiments. The results were subjected to statistical analysis by t-test, p* < 0.05, after three independent experiments.

**Suppl.Fig.16**. The body weight of all mice used for the treatment of taxol and USP24-i was detected.

**Suppl**.**Fig.17.** Lung cancer cell line, T24, was injected into SCID mice, then treated with Taxol and NCI677397 two time per week for two weeks. The levels of various cancer stemness markers were studied by IHC with indicated antibodies.

**Suppl.Fig.18.** The effects of NCI677397 (A) and NCI158067 (B) in lung cancer migratory ability were studied by Wound healing assay. The cytotoxicity of taxol in T24 cells with or without NCI158067 treatment (C). The results were subjected to statistical analysis by t-test, p* < 0.05, p*** < 0.005, after three independent experiments.

**Suppl.Fig.19.** The effects of NCI677397 and NCI158067 in A549 cells proliferation (A) and cell migratory activity (B). The results were subjected to statistical analysis by t-test, p** < 0.01, p***<0.005, after three independent experiments.

**Suppl.Fig.20.** GFP or GFP-USP24 were overexpressed in A549 cells, and treated with short time UV exposure. After 3 h recovery, the level of γ-H2AX was studied by IF with anti-γ-H2AX antibodies.
