# Supplementary figures and images for: USP24 promotes drug resistance during cancer therapy
Source: Cell Death Differ. 2021 Apr 12;28(9):2690–707. doi: 10.1038/s41418-021-00778-z (PMC8408266; doi:10.1038/s41418-021-00778-z)

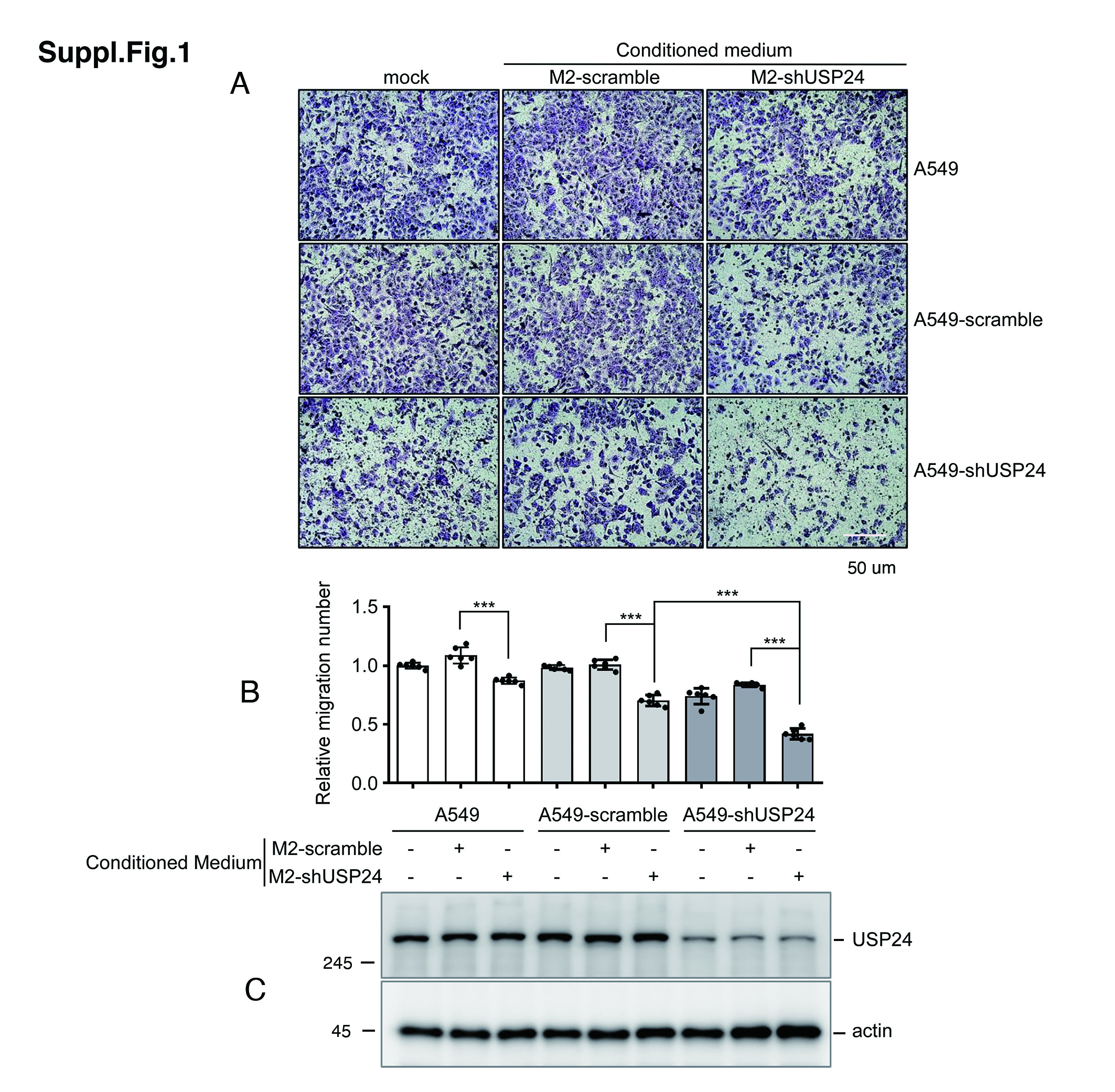

Supplement: Supplementary file 2 — Supplementary Fig.1 [file 41418_2021_778_MOESM2_ESM.tif]

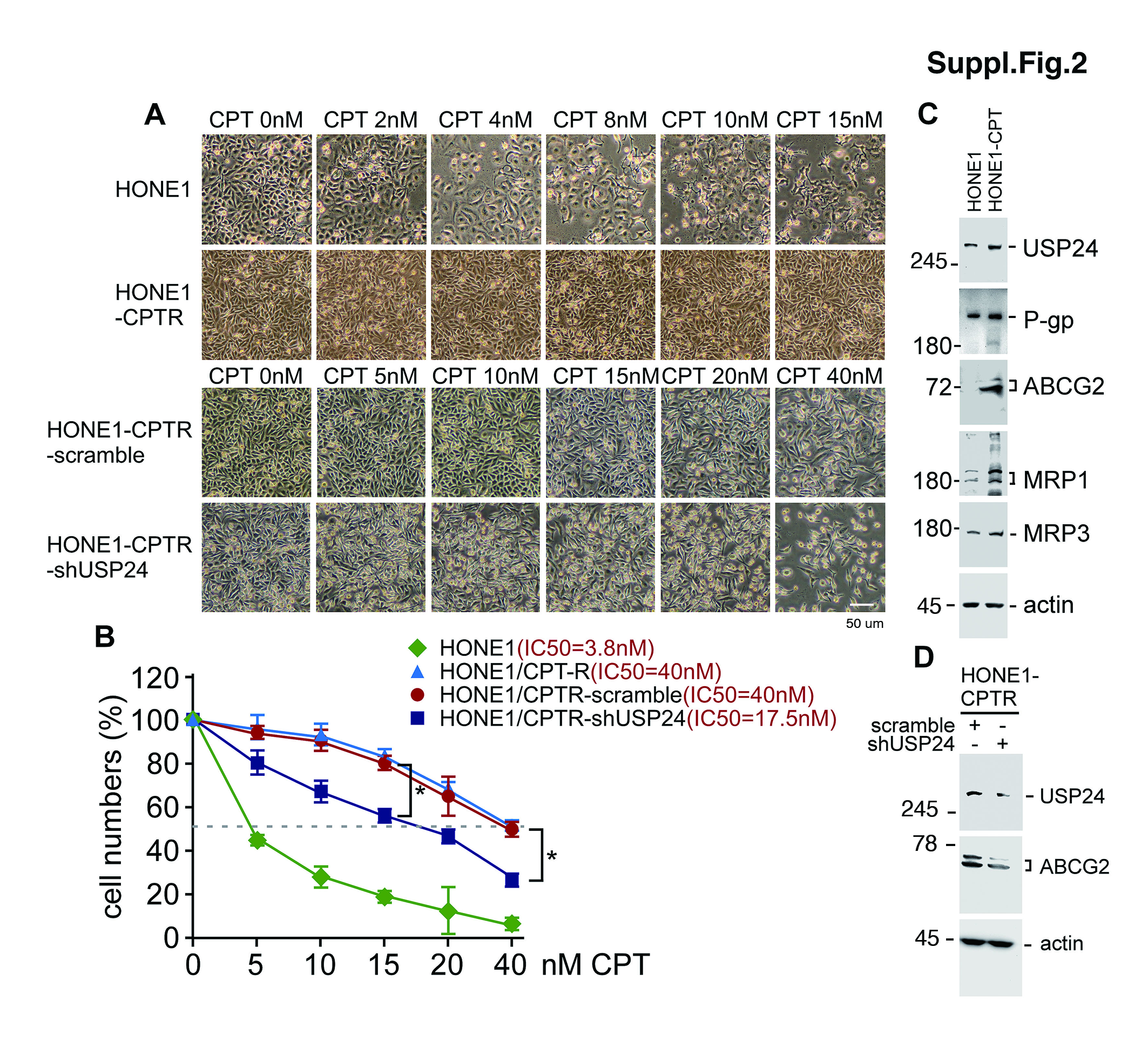

Supplement: Supplementary file 3 — Supplementary Fig.2 [file 41418_2021_778_MOESM3_ESM.tif]

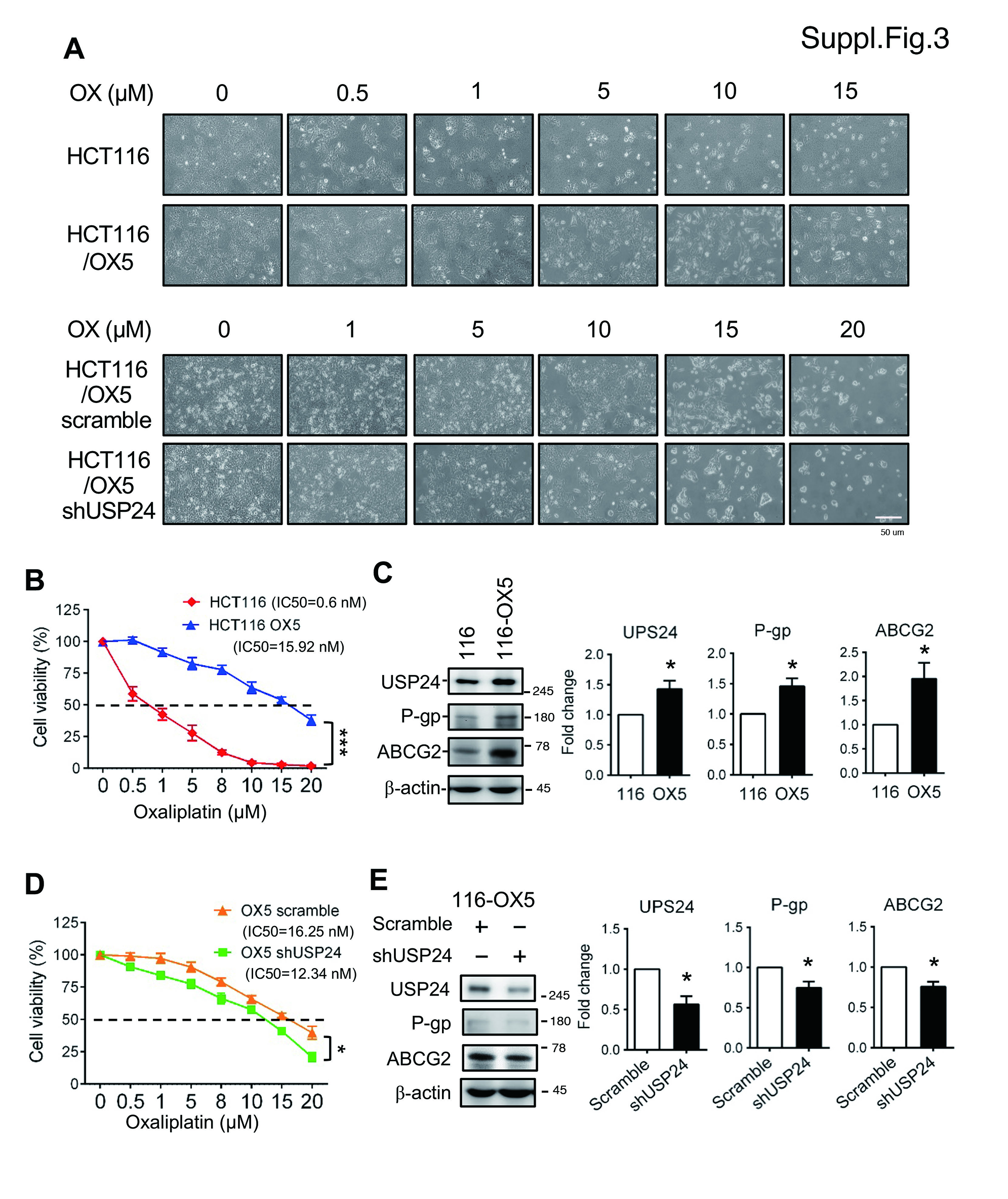

Supplement: Supplementary file 4 — Supplementary Fig.3 [file 41418_2021_778_MOESM4_ESM.tif]

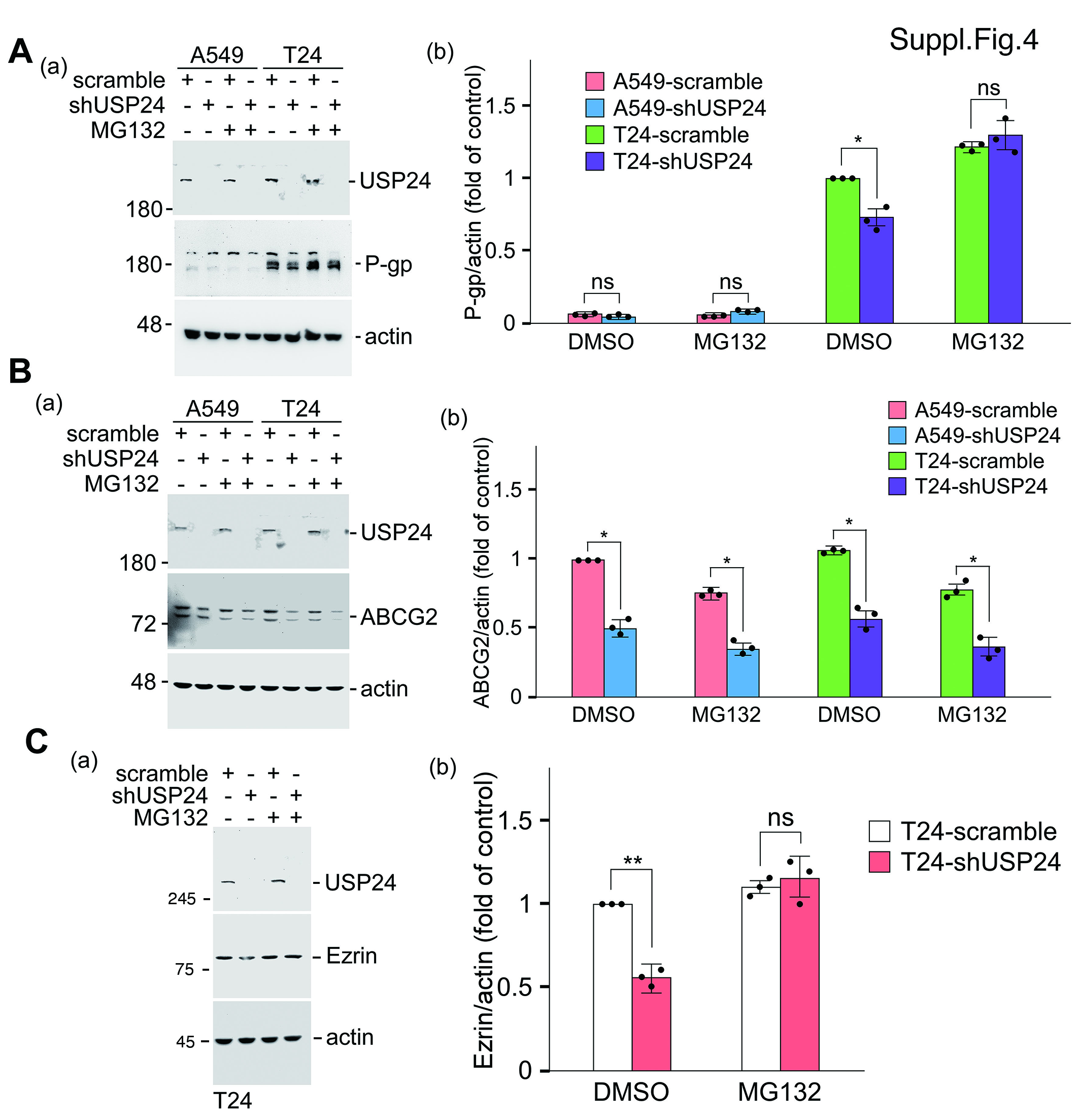

Supplement: Supplementary file 5 — Supplementary Fig.4 [file 41418_2021_778_MOESM5_ESM.tif]

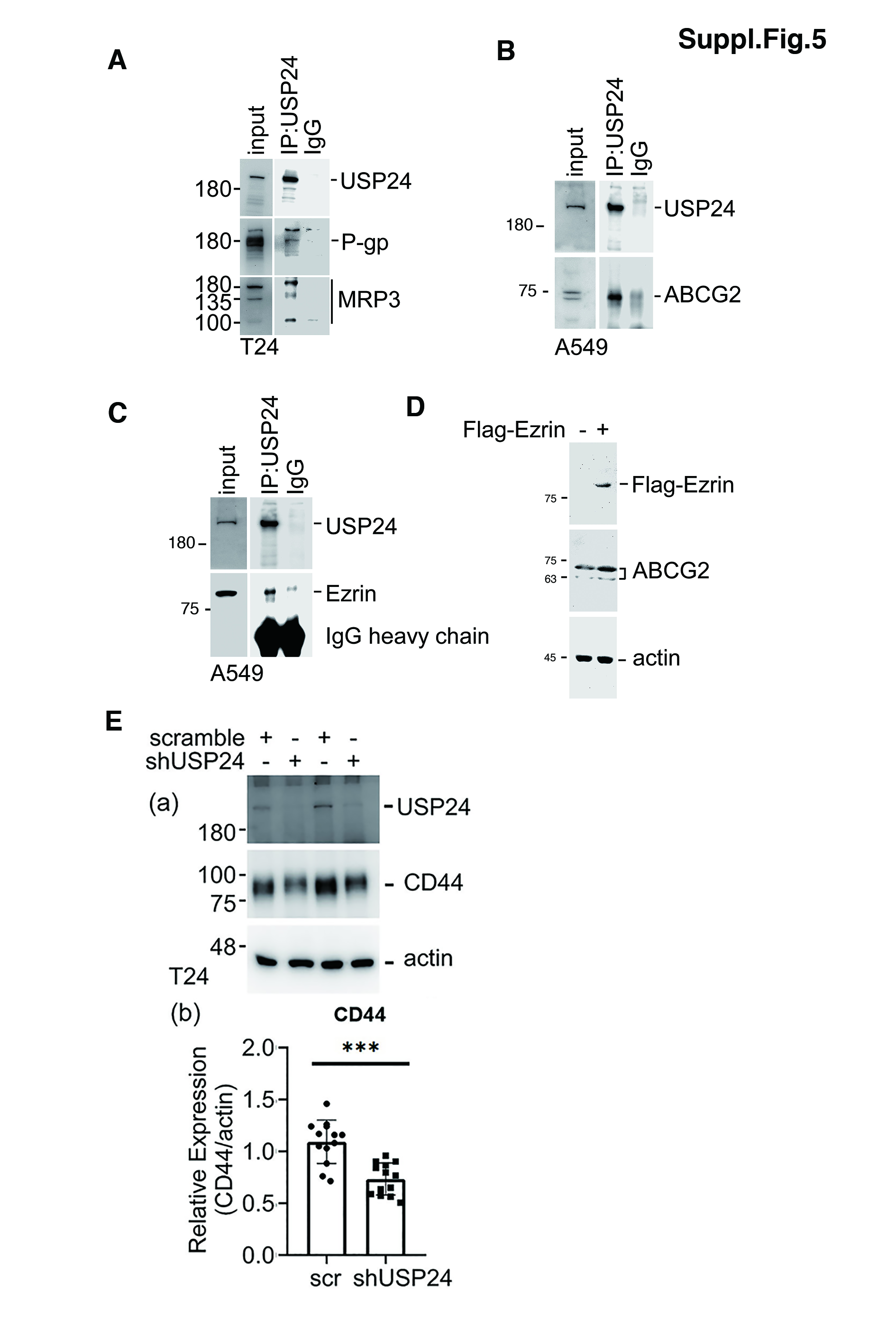

Supplement: Supplementary file 6 — Supplementary Fig.5 [file 41418_2021_778_MOESM6_ESM.tif]

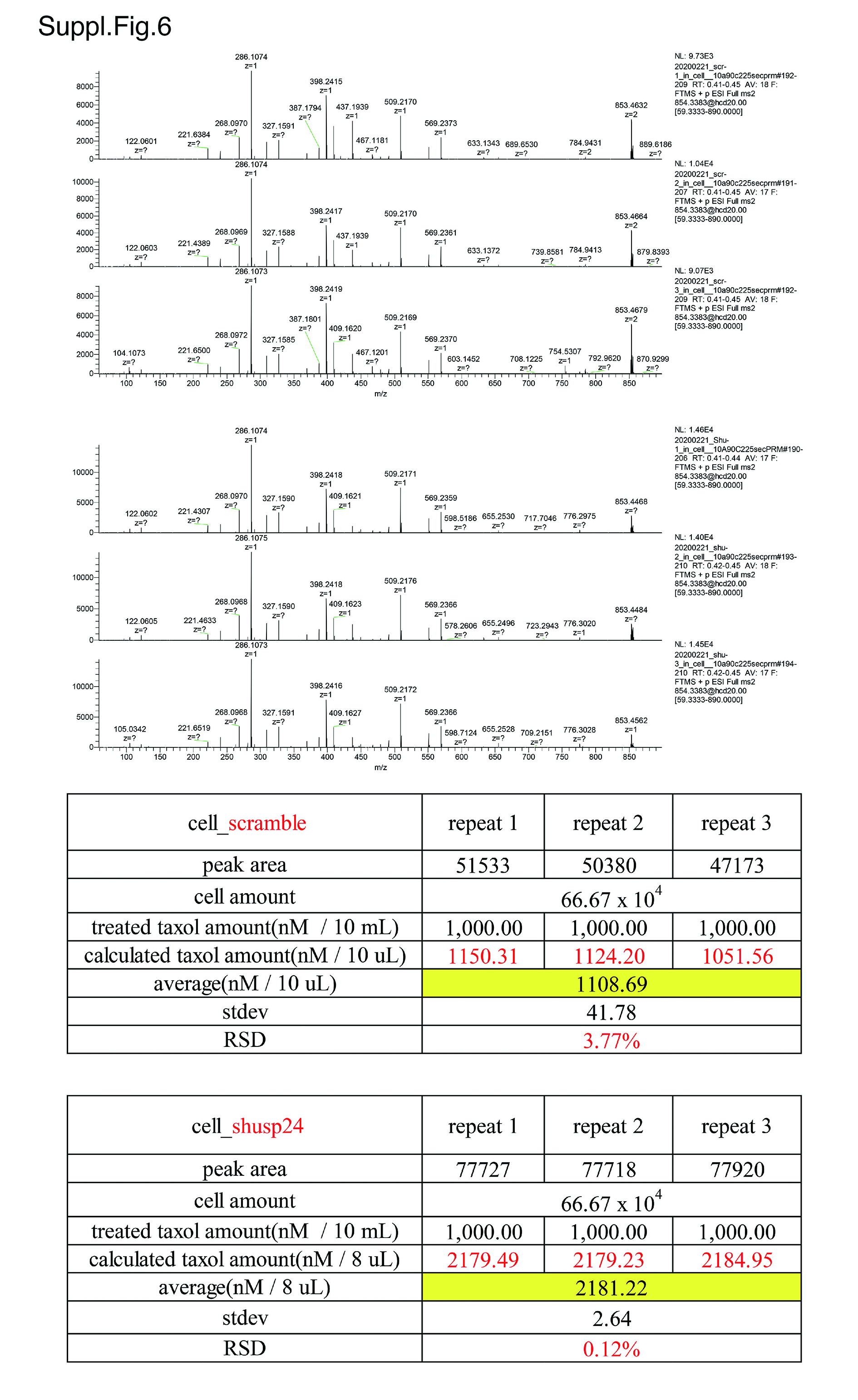

Supplement: Supplementary file 7 — Supplementary Fig.6 [file 41418_2021_778_MOESM7_ESM.tif]

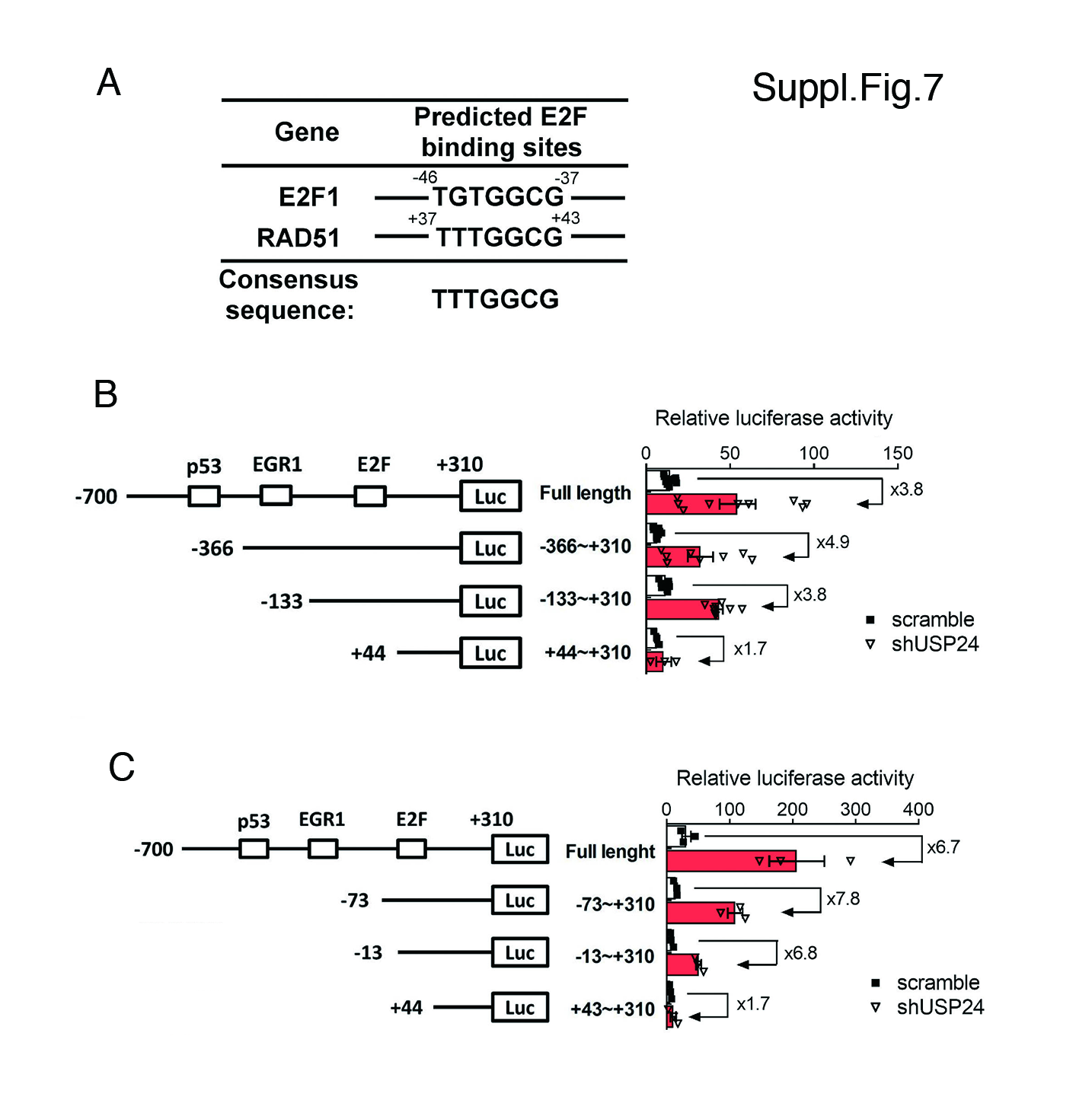

Supplement: Supplementary file 8 — Supplementary Fig.7 [file 41418_2021_778_MOESM8_ESM.tif]

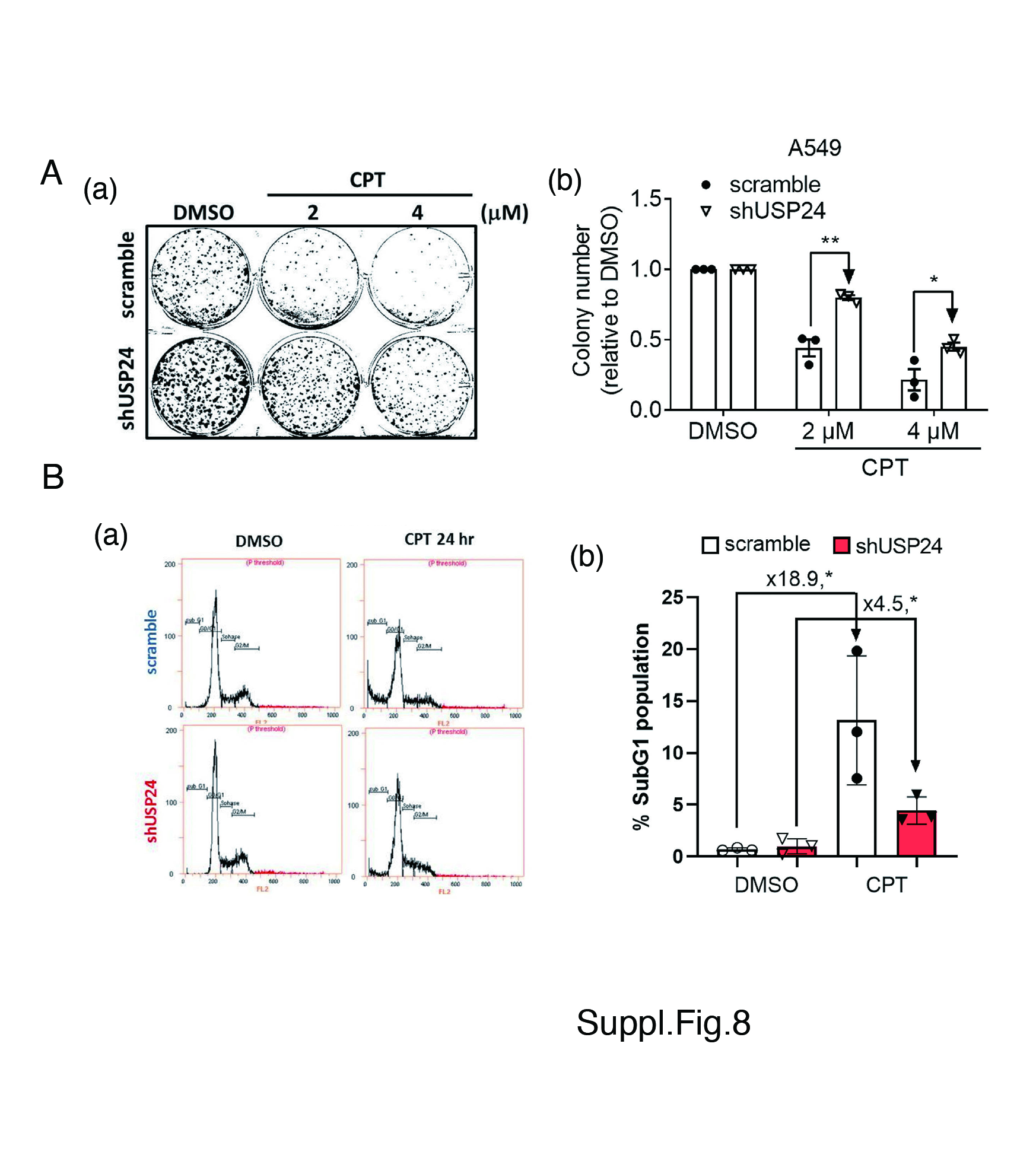

Supplement: Supplementary file 9 — Supplementary Fig.8 [file 41418_2021_778_MOESM9_ESM.tif]

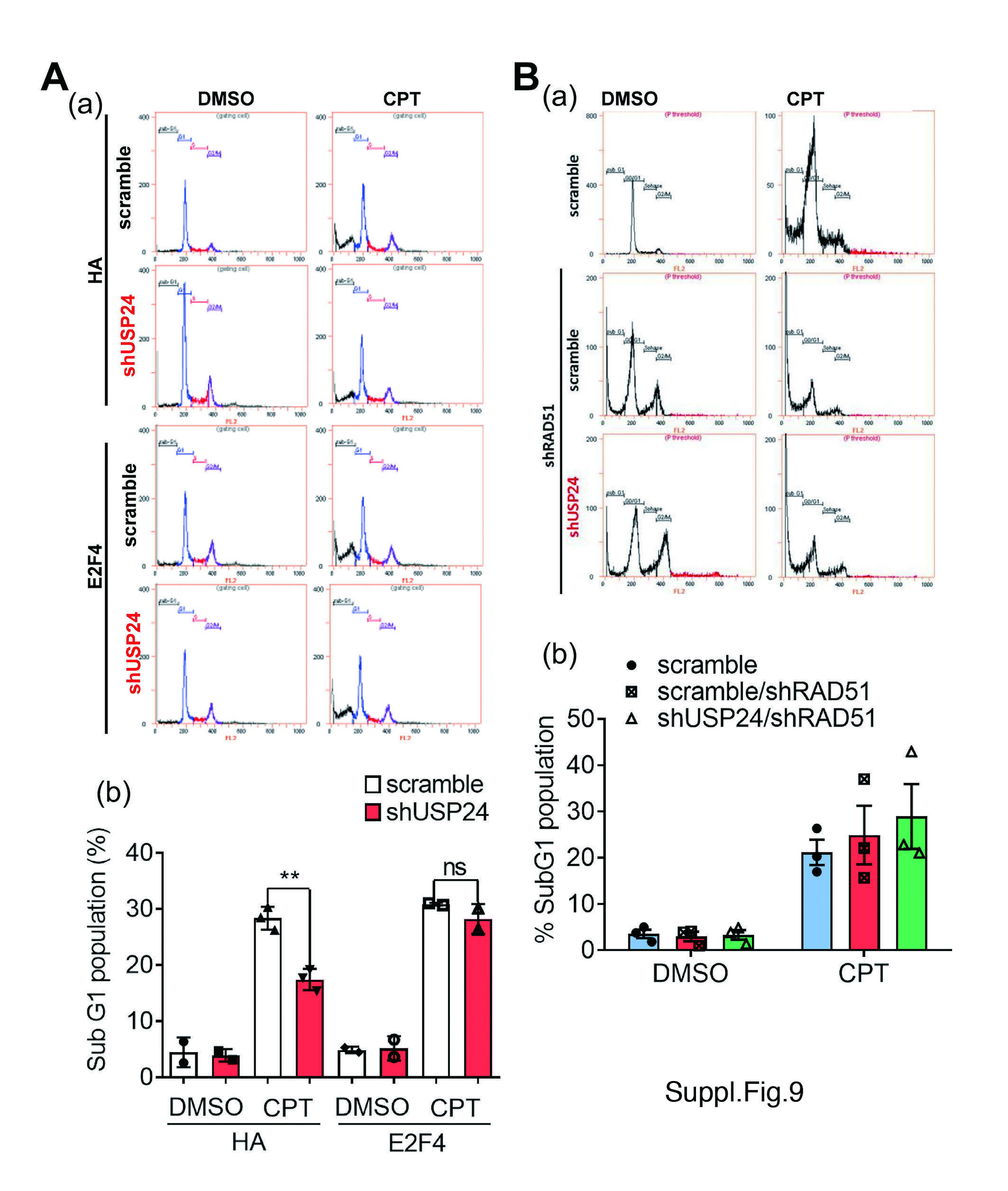

Supplement: Supplementary file 10 — Supplementary Fig.9 [file 41418_2021_778_MOESM10_ESM.jpg]

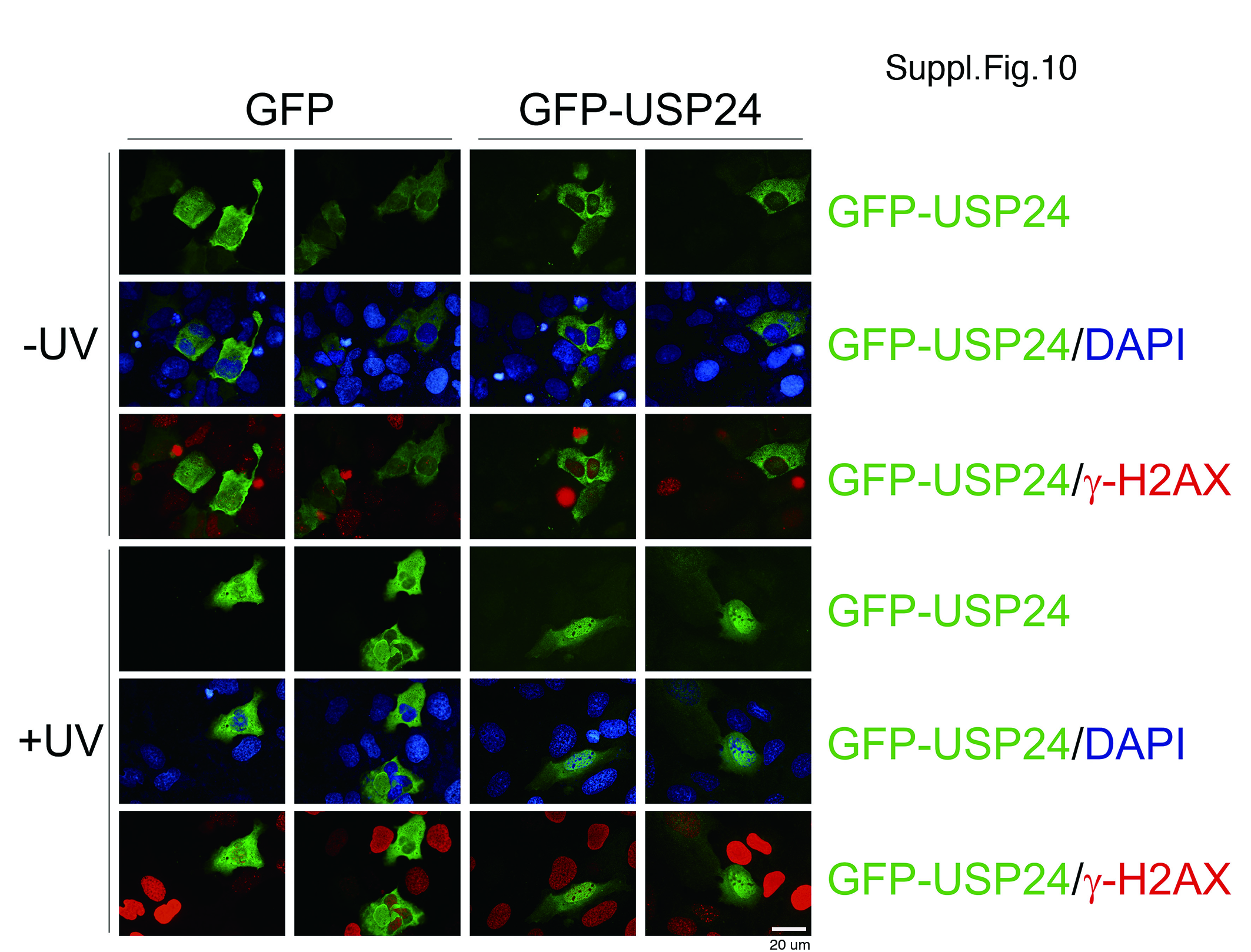

Supplement: Supplementary file 11 — Supplementary Fig.10 [file 41418_2021_778_MOESM11_ESM.tif]

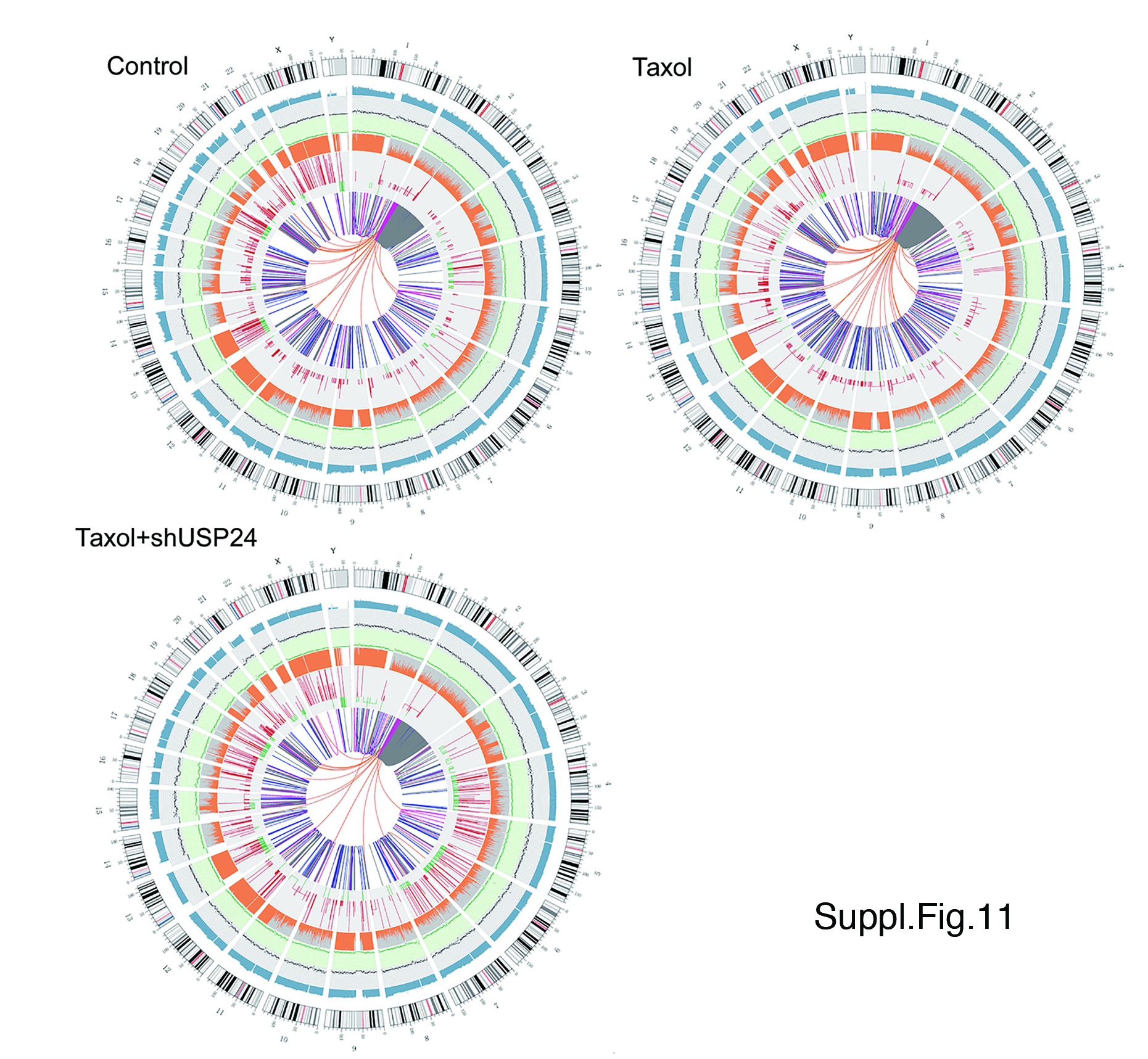

Supplement: Supplementary file 12 — Supplementary Fig.11 [file 41418_2021_778_MOESM12_ESM.tif]

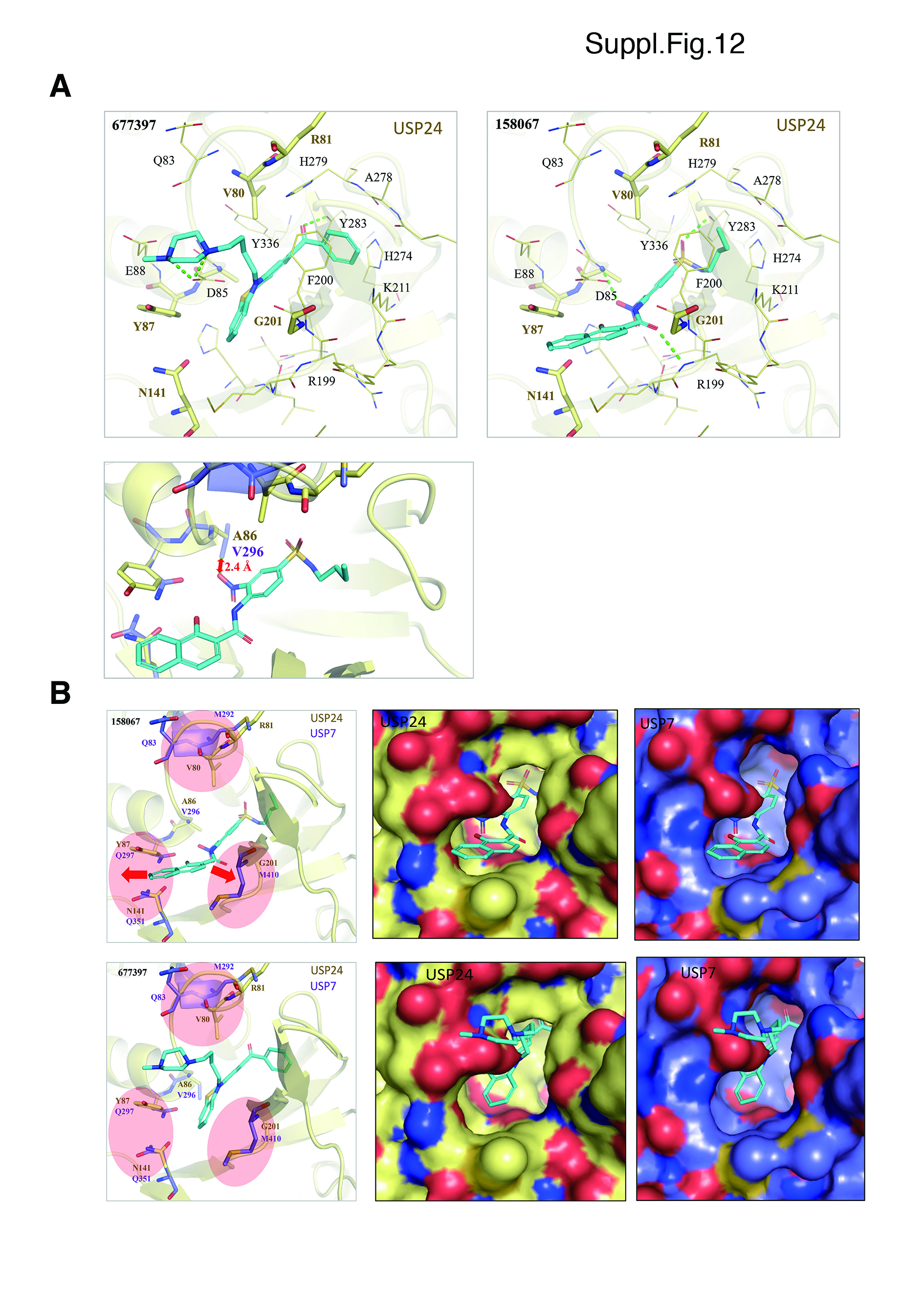

Supplement: Supplementary file 13 — Supplementary Fig.12 [file 41418_2021_778_MOESM13_ESM.tif]

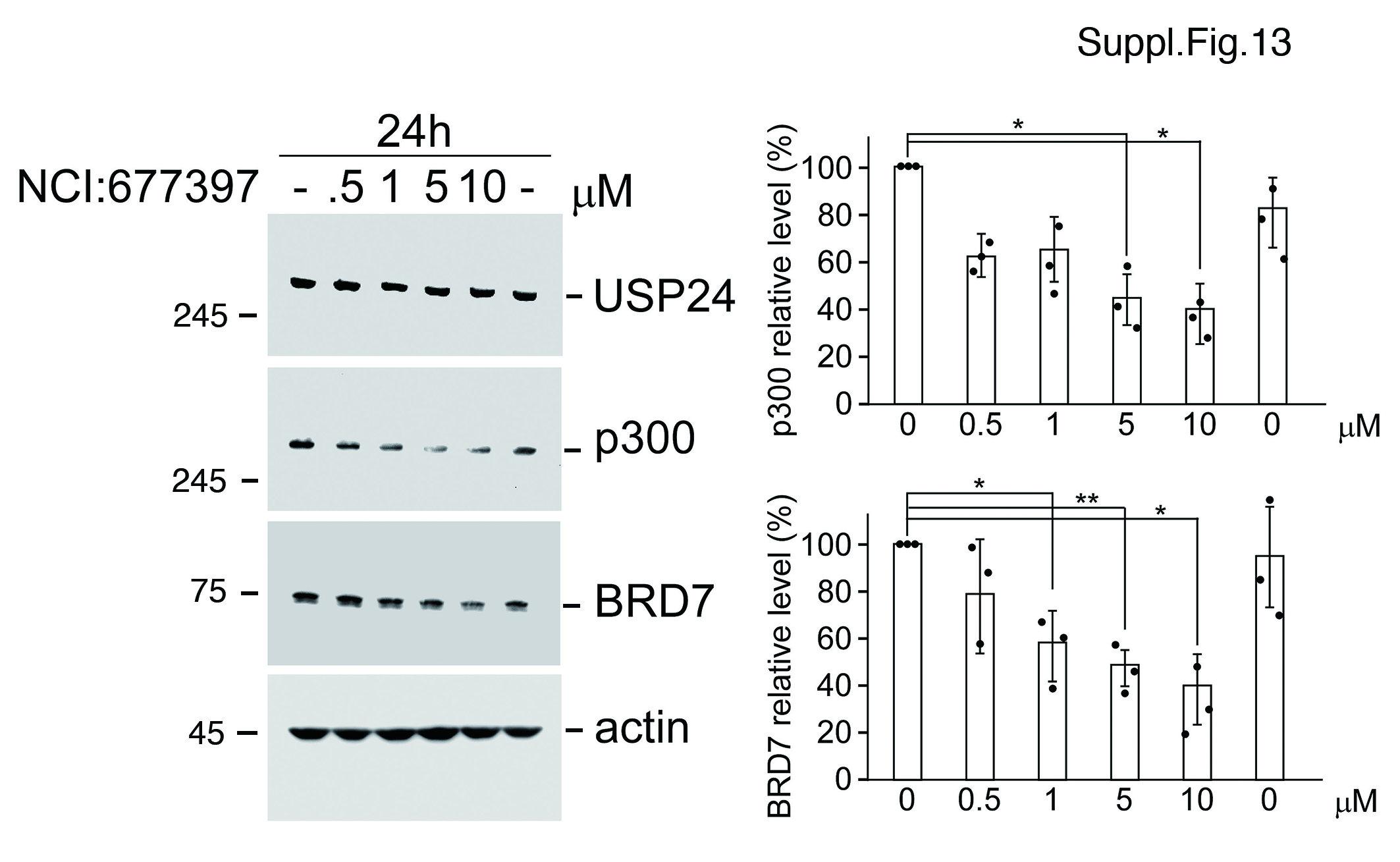

Supplement: Supplementary file 14 — Supplementary Fig.13 [file 41418_2021_778_MOESM14_ESM.tif]

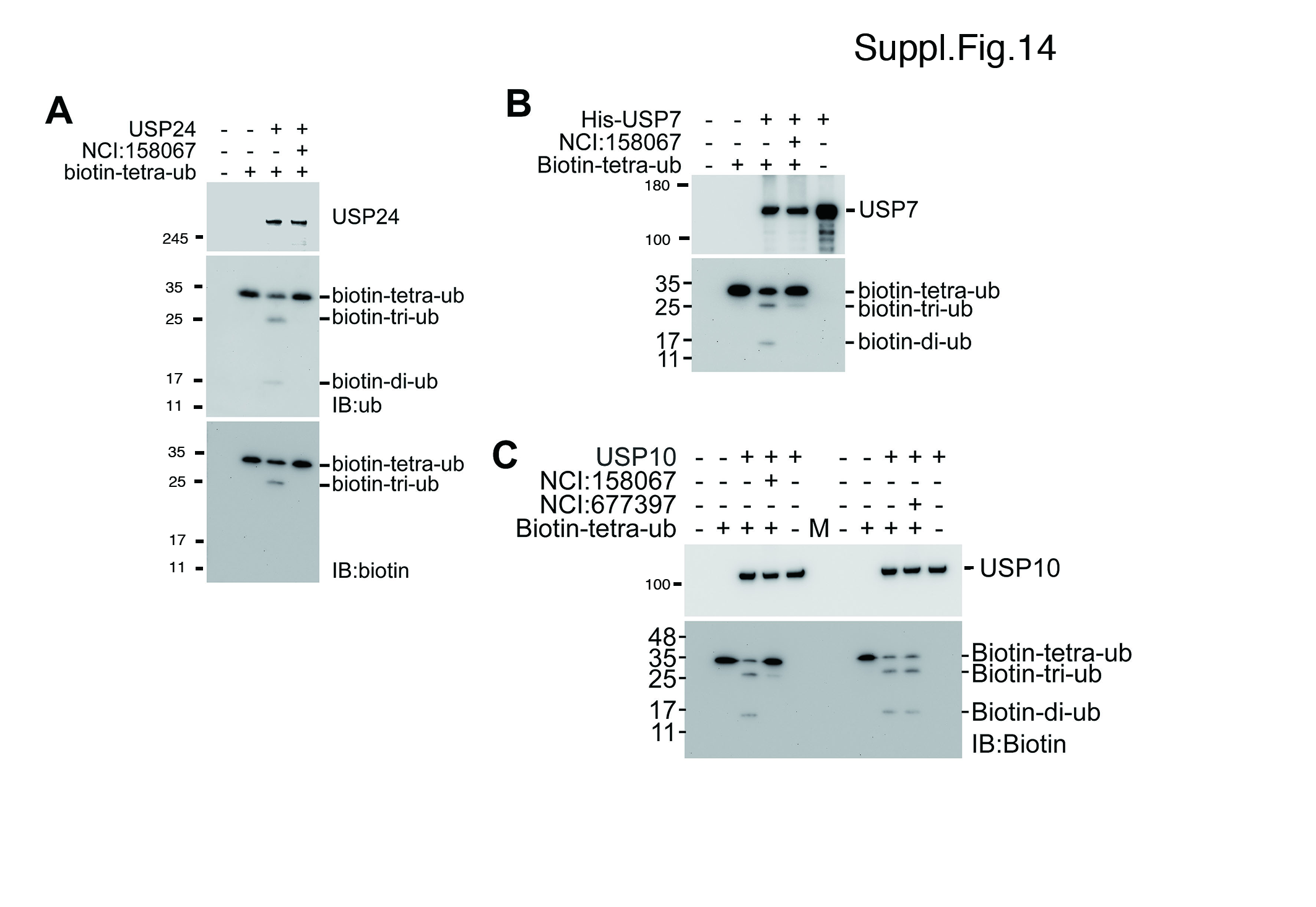

Supplement: Supplementary file 15 — Supplementary Fig.14 [file 41418_2021_778_MOESM15_ESM.tif]

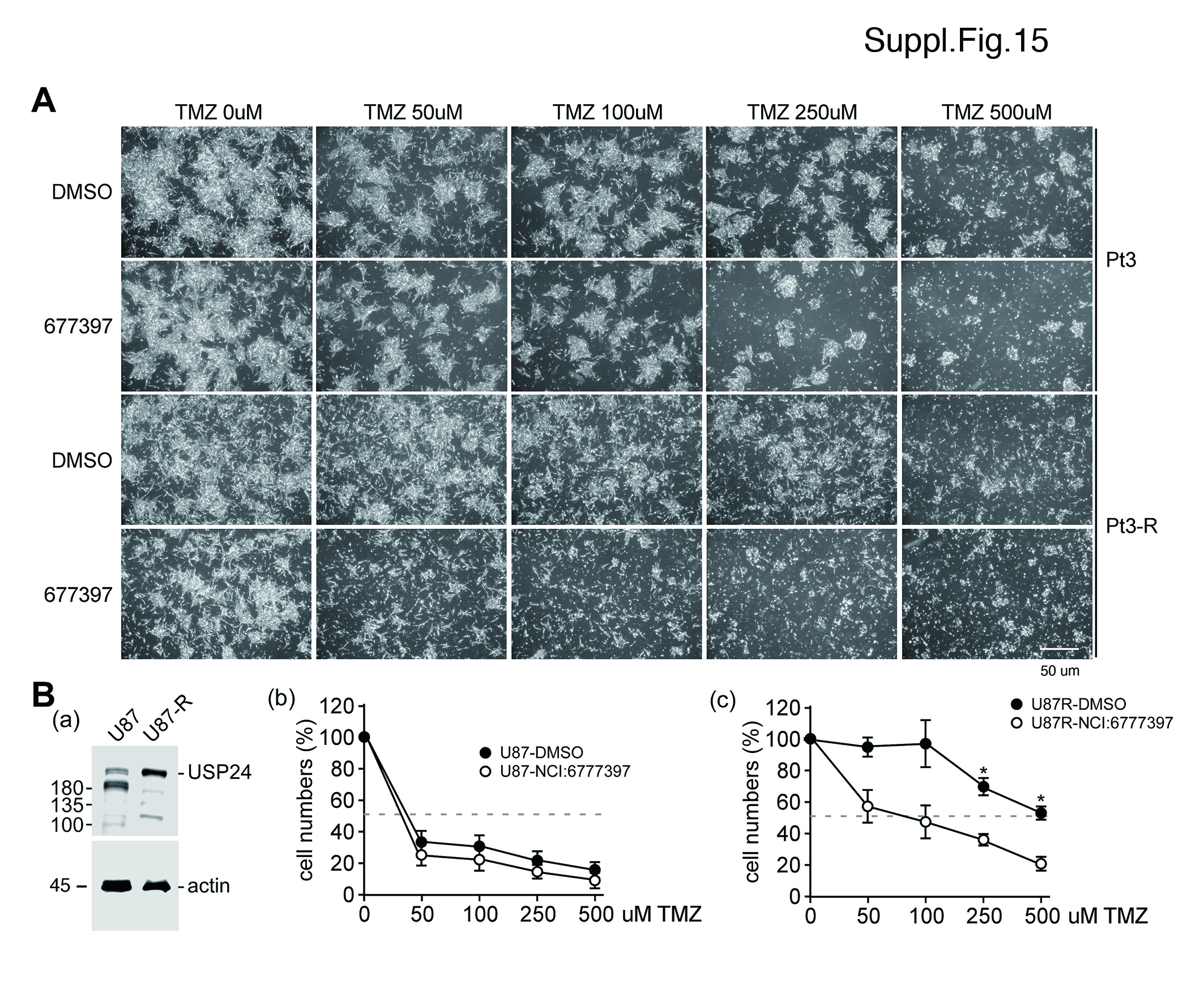

Supplement: Supplementary file 16 — Supplementary Fig.15 [file 41418_2021_778_MOESM16_ESM.tif]

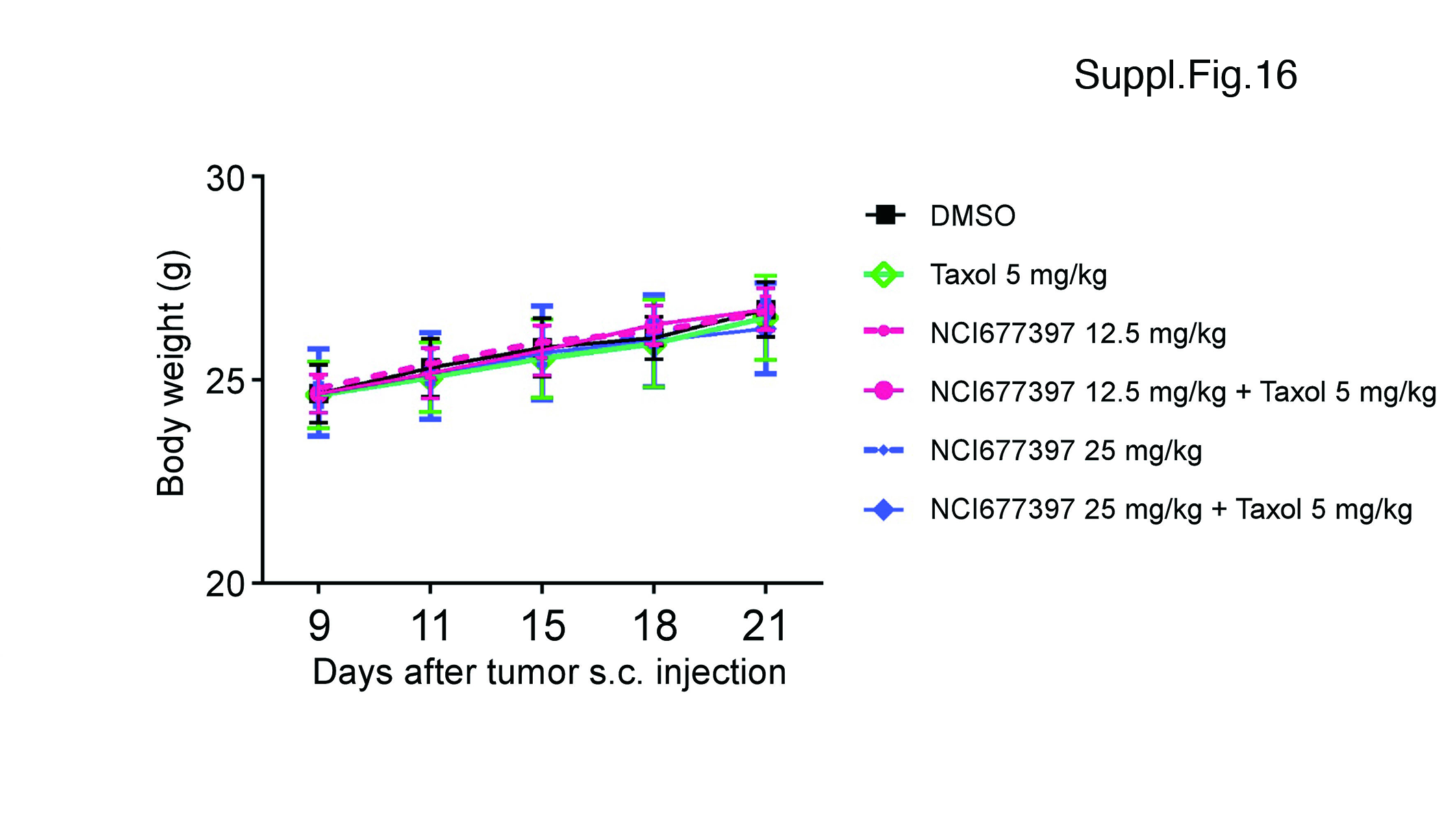

Supplement: Supplementary file 17 — Supplementary Fig.16 [file 41418_2021_778_MOESM17_ESM.tif]

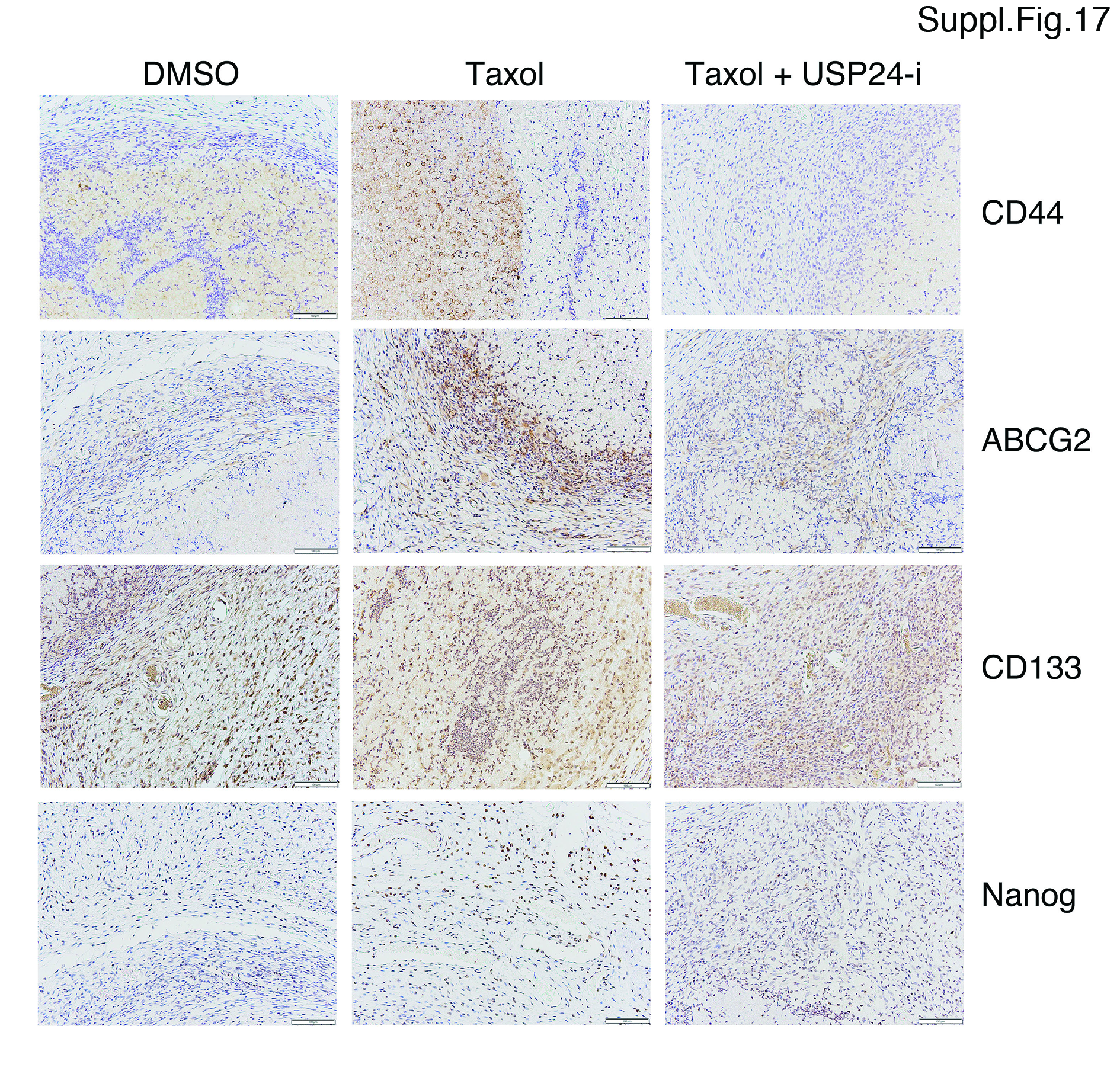

Supplement: Supplementary file 18 — Supplementary Fig.17 [file 41418_2021_778_MOESM18_ESM.tif]

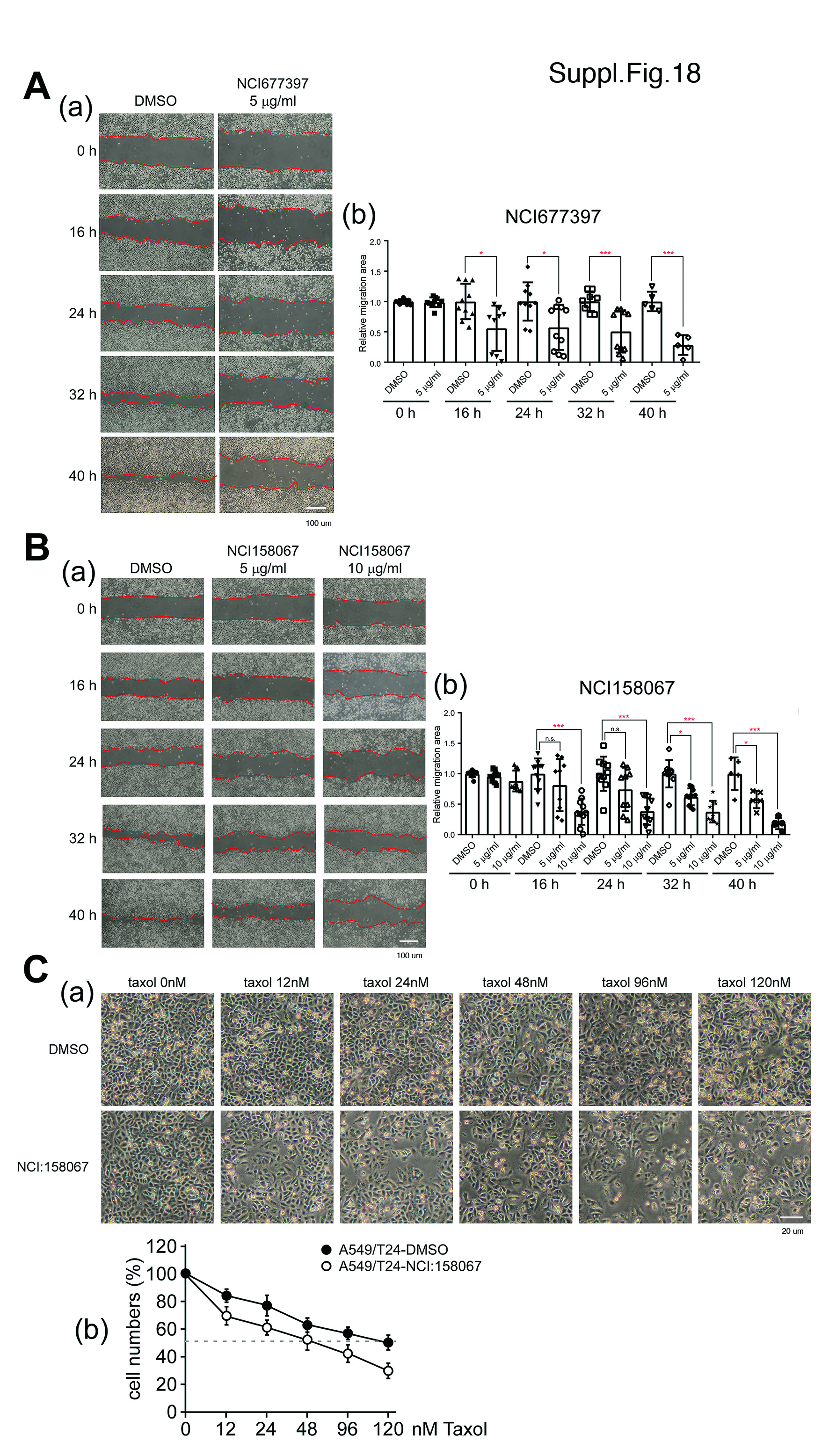

Supplement: Supplementary file 19 — Supplementary Fig.18 [file 41418_2021_778_MOESM19_ESM.tif]

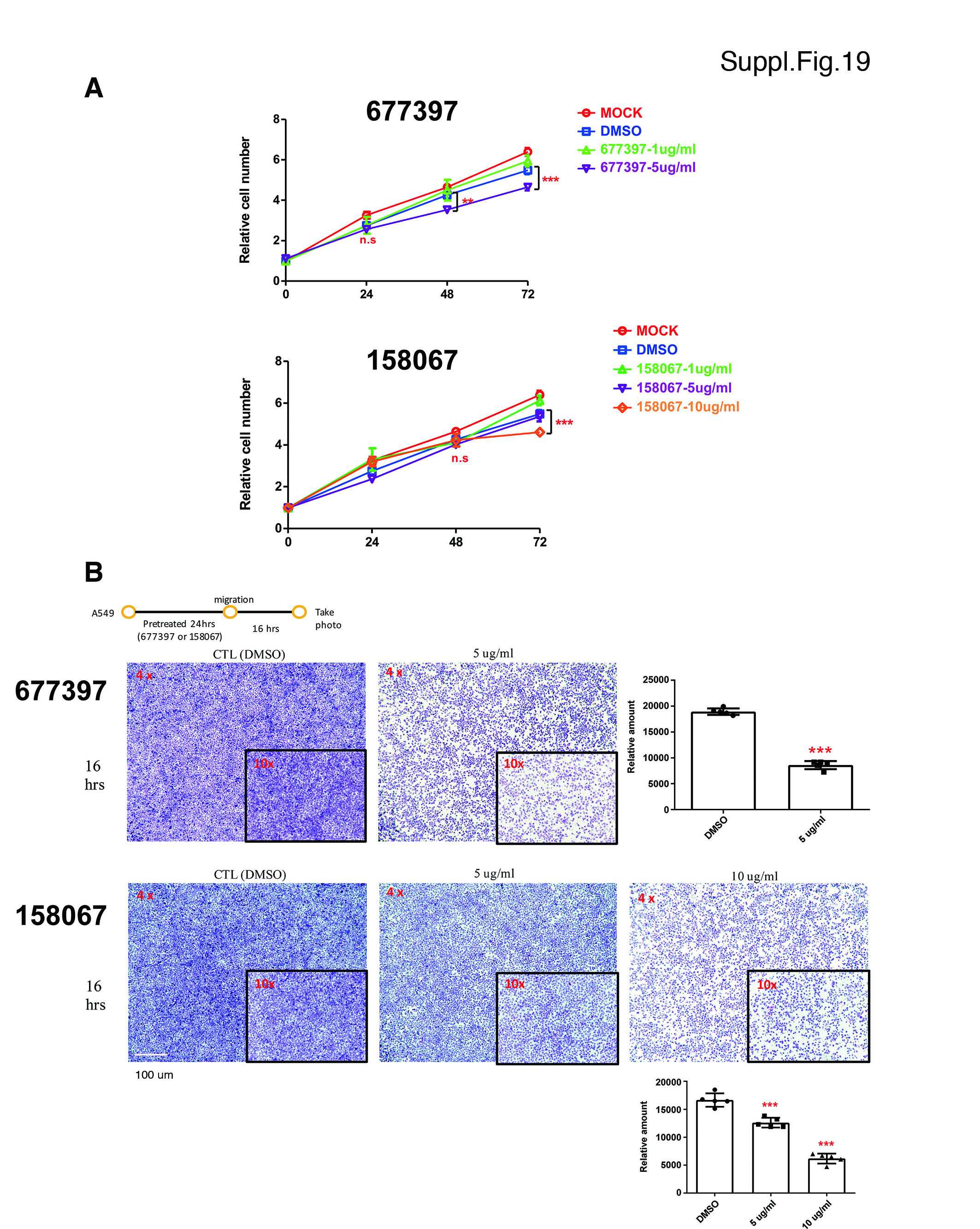

Supplement: Supplementary file 20 — Supplementary Fig.19 [file 41418_2021_778_MOESM20_ESM.tif]

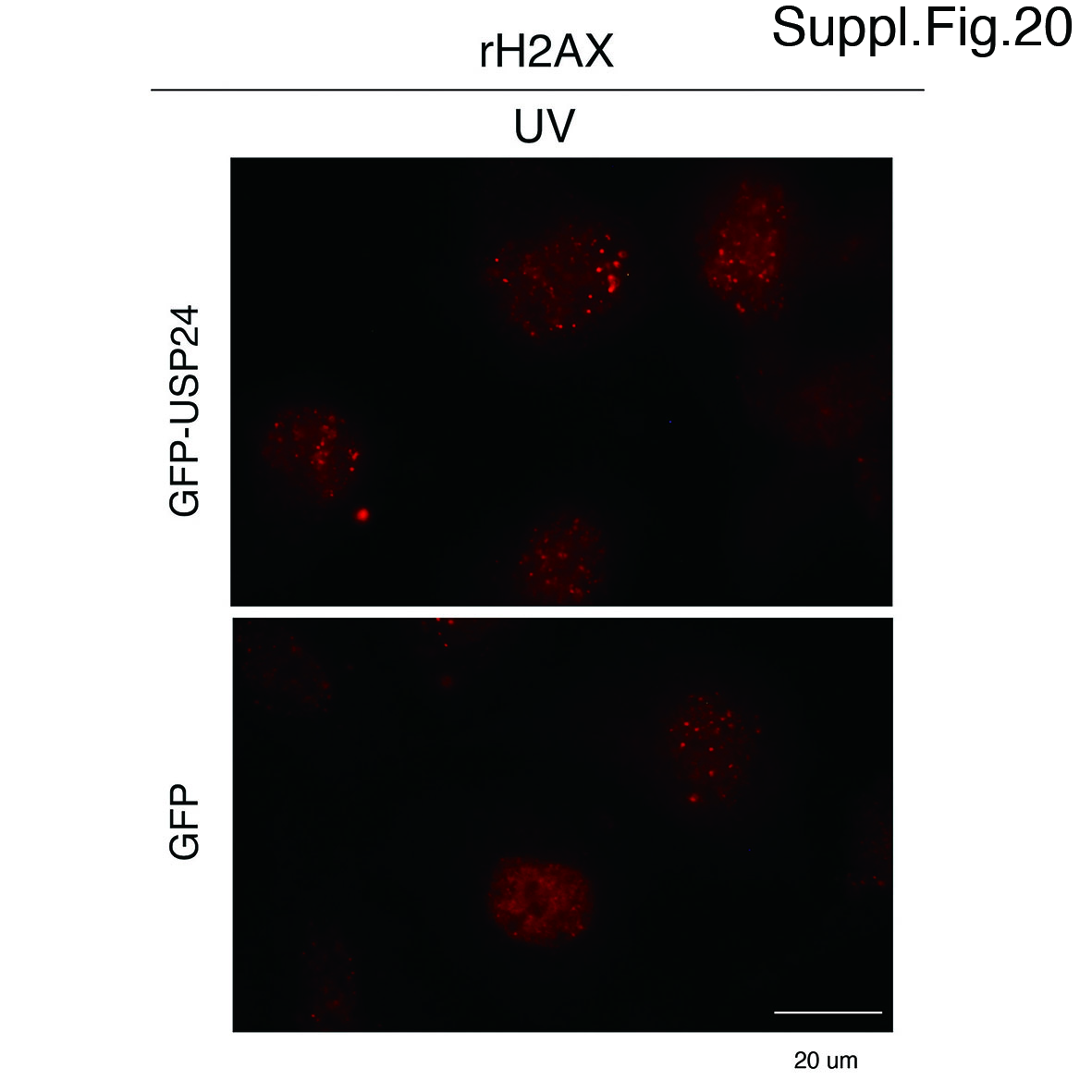

Supplement: Supplementary file 21 — Supplementary Fig.20 [file 41418_2021_778_MOESM21_ESM.tif]
